# Targeting transcription-replication conflicts using G-quadruplexes stabilizers in multiple myeloma

**DOI:** 10.1016/j.bneo.2025.100072

**Published:** 2025-01-20

**Authors:** Laure Dutrieux, Sara Ovejero, Antoine Guillemin, Miss Leriem Zellagui, Elke De Bruyne, Catharina Muylaert, Lien Van Hemelrijck, Yea-Lih Lin, Elle Loughran, Armelle Choquet, Talha Magat, Soumya Bouchouika, Caroline Bret, Guilhem Requirand, Nicolas Robert, Laure Vincent, Guillaume Cartron, Charles Herbaux, Raphaël Rodriguez, Michel Cogné, Eric Rivals, Jean-Christophe Andrau, Alexandre David, Philippe Pasero, Jérôme Moreaux

**Affiliations:** 1 Institute of Human Genetics, Unité Mixte de Recherche Centre National de la Recherche Scientifique Université de Montpellier 9002, Montpellier, France; 2 Department of Biological Hematology, Centre Hospitalier Universitaire Montpellier, Montpellier, France; 3 Department of Hematology and Immunology, Myeloma Center Brussels, Vrije Universiteit Brussel, Brussels, Belgium; 4 Translational Oncology Research Center, Vrije Universiteit Brussel, Brussels, Belgium; 5 Smurfit Institute of Genetics, Trinity College, Dublin, Ireland; 6 Laboratoire d'informatique, de robotique et de microélectronique de Montpellier, University of Montpellier, Centre National de la Recherche Scientifique, Montpellier, France; 7 Institut de Recherche en Cancérologie de Montpellier, University of Montpellier, Institut Régional du Cancer de Montpellier, INSERM, Montpellier, France; 8 Institut de Génétique Moléculaire de Montpellier, University of Montpellier, Centre National de la Recherche Scientifique- Unité Mixte de Recherche, 5535, Montpellier, France; 9 Department of Clinical Hematology, Centre Hospitalier Universitaire Montpellier, Montpellier, France; 10 University of Montpellier, UFR Medicine, Montpellier, France; 11 Institut Curie, Centre National de la Recherche Scientifique, INSERM, PSL Research University, Equipe Labellisée Ligue Contre le Cancer, Paris, France; 12 Institut National de La Santé et de La Recherche Médicale, Unité Mixte de Recherche U1236, Université de Rennes, Etablissement Français Du Sang Bretagne, Rennes, France; 13 Centre Hospitalier Universitaire de Rennes, Suivi Immunologique des Thérapies Innovantes, Pôle Biologie, Rennes, France; 14 IRMB-PPC, INM, University of Montpellier, Centre Hospitalier Universitaire Montpellier, INSERM Centre National de la Recherche Scientifique, Montpellier, France; 15 Institut Universitaire de France, Paris, France

## Abstract

Replication stress exerts an important role in fueling genomic instability characterizing multiple myeloma (MM) evolution and is a leading cause of drug resistance. Normal and malignant plasma cells (PCs) are associated with a high transcriptional stress due to the huge production of immunoglobulins. Transcription-replication conflicts (TRCs), arising from collisions between replication and transcription machineries, can promote tumor progression and represent an Achilles’ heel to cancer cells. We reported a gene signature related to TRCs management (TRC score), overexpressed in malignant vs normal PCs. High TRC score identified patients with MM with a poor prognosis who could benefit from a TRC-enhancing therapy, in independent cohorts of patients with MM treated with high-dose melphalan chemotherapy or anti-CD38 immunotherapy. Here, we investigated the therapeutic interest of increasing TRCs to target specifically malignant PCs using the G-quadruplex (G4) stabilizer pyridostatin (PDS). PDS exerted significant toxicity in MM cell lines and primary MM cells, inducing DNA damage, cell cycle arrest, and apoptosis. Importantly, primary myeloma cells are significantly more sensitive to PDS treatment than normal bone marrow cells. Moreover, PDS improved the efficacy of MM treatments such as melphalan and histone deacetylase (HDAC) or bromodomain (BRD) inhibitors. Thus, our study shows that G4 stabilizers could be used to specifically target MM cells that exhibit concomitant replication stress and a high level of transcription, through the increase of TRCs. These molecules could be used to increase the efficacy of other treatments including melphalan, HDAC inhibitors, and BRD inhibitors.

## Key Points

• High-risk patients with MM overexpressed genes involved in TRC resolution which suggests that they could benefit from a TRC-enhancing therapy.
• Increasing TRCs with G4 stabilizers increases the efficacy of current MM treatments such as melphalan and HDAC or BRD inhibitors.

## Visual Abstract

**Figure:**
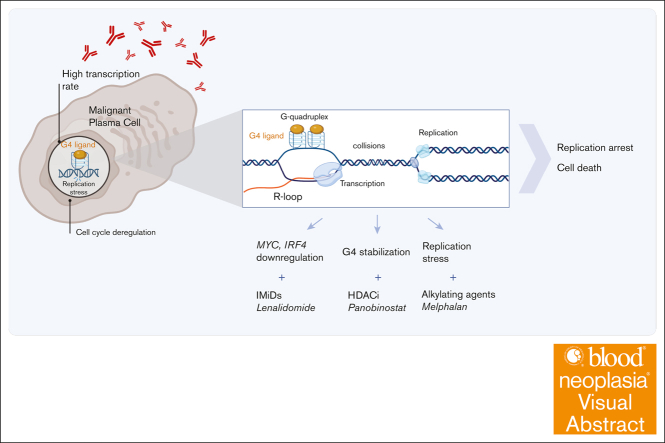

## Introduction

Multiple myeloma (MM) is the second-most frequent blood cancer, characterized by clonal proliferation of terminally differentiated plasma cells (PCs) in the bone marrow (BM).^1^ Despite considerable progress in the treatment of MM, particularly with the emergence of immune-based therapies, it remains unclear how to overcome drug resistance, especially in high-risk patients with *TP53* abnormalities, including del17p and/or *TP53* mutations.^2^, ^3^, ^4^ Deregulated oncogene expression can alter replication timing and transcriptional programs, resulting in increased replication stress (RS), generally referred to as oncogene-induced RS.^5^ Accordingly, MM cells (MMCs) but not normal PCs exhibit constitutive phosphorylation of the RS marker H2A.X (termed γH2A.X).^6^ Oncogene overexpression also results in hyperactivation of the transcriptional machinery required to sustain increased proliferation and cell growth.^7^^,^^8^ Hypertranscription thus exacerbates RS by impeding replication fork progression, often resulting in transcription-replication conflicts (TRCs). MMCs must cope with both oncogene-induced RS and a high level of transcription linked to immunoglobulin synthesis, thus constituting a positive loop that sustains genomic instability. In line with this, increased transcription in MM has been associated with increased mutation rate.^9^ This genomic instability is therefore an Achilles’ heel for MMCs, which are expected to be more dependent on the DNA damage response (DDR) for survival than normal PCs, offering exploitable targets for the development of new therapeutic strategies.

Non–B DNA structures such as G-quadruplexes (G4s) and R-loops can promote TRCs and RS by constituting obstacles for the replication forks.^10^ G4s are highly stable structures found in G-rich regions of the DNA and formed by stacking of guanine tetrads stabilized by Hoogsteen hydrogen bonds and monovalent cations.^11^ They are located in key regulatory regions, such as promoters, gene bodies, enhancers, telomeres, or *IGH* locus recombination sites, and involved in the regulation of numerous biological processes such as DNA replication, transcription, or epigenetic regulation. Interestingly, they are overrepresented in the promoter of many proto-oncogenes, such as *MYC*, *BCL2*, or *KRAS*, which has prompted the development of small molecules stabilizing these structures to alter oncogene transcription.^12^^,^^13^ Such small molecules are called G4 stabilizers or G4 ligands, and their mechanisms of action involve not only oncogene downregulation but also DNA damage.^14^^,^^15^ RS induced by these molecules could also involve R-loop stabilization, because G4s can form in the displaced DNA strand comprised in an R-loop.^10^^,^^16^ Even though no such stabilizer is currently approved for cancer treatment, they are progressing in clinical trials and a growing body of evidence demonstrates their therapeutic potential.

Here, we developed a gene expression–based score showing that high expression of a TRC-resolution machinery was associated with poor outcomes in patients with MM. The G4 stabilizer pyridostatin (PDS) showed significant toxicity against human myeloma cell lines (HMCLs) as well as primary MMCs. PDS-induced transcription-dependent DNA damage, cell cycle arrest, and apoptosis. PDS synergized with current MM treatments, such as melphalan, lenalidomide, and panobinostat, and with new therapies such as bromodomain inhibitors (BRDis). Altogether, these results show the importance of TRC management in MM and demonstrate that targeting oncogene-induced RS and hypertranscription with G4 stabilizers could be a new therapeutic strategy for patients with MM.

## Methods

### Gene expression analysis

Affymetrix data from independent cohorts of newly diagnosed patients with MM treated with high-dose therapy (HDT) and autologous hematopoietic stem cell transplantation (ASCT) were used to develop the TRC score. TT2 cohort was used as the training cohort (University of Arkansas for Medical Sciences, Little Rock, AR; n = 345; Gene Expression Omnibus GSE2658). Publicly available cohorts of newly diagnosed patients with MM treated with HDT and ASCT (CoMMpass [NCT01454297; version IA11a], HM [Heidelberg/Montpellier; n = 206, E-MTAB-372], and Hovon [GSE19784] cohorts) and a cohort of patients at relapse treated with the anti-CD38 monoclonal antibody (mAb) daratumumab (Montpellier cohort) were also used for validation.

### HMCLs and primary MM samples

XGs cell lines were obtained as previously described.^17^ AMO1, LP1, L363, SKMM2, KMS27, MOLP8, and OPM2 were purchased from DSMZ (Braunsweig, Germany) and RPMI8226 from ATCC (Rockville, MD). JJN3 was kindly provided by Van Riet (Bruxelles, Belgium). These cell lines were maintained in RPMI1640 GlutaMAX (61870044, Gibco) supplemented with 10% fetal bovine serum (CVFSVF0001, Eurobio) and with interleukin-6 (IL-6; 2 ng/mL) for the XGs. HMCLs were authenticated according to their short tandem repeat profiling and gene expression profiling using Affymetrix U133 plus 2.0 microarrays deposited in the ArrayExpress public database (accession numbers E-TABM-937 and E-TABM-1088).

All primary BM samples were collected with the approval of the institutional research board of Montpellier University Hospital (DC2008-417) after patients’ written informed consent, in accordance with the Declaration of Helsinki. Mononuclear cells were isolated from total BM using Ficoll density gradient centrifugation (Nycomed, Zurich, Switzerland) and cultured in the presence of 2 ng/mL IL-6. Ninety-six hours after treatment with increasing concentrations of PDS, panobinostat, or I-BET-762, the percentage of CD138^+^ CD38^+^ PCs and CD138^–^ nonmyeloma cells were determined by flow cytometry as previously described.^18^

### Cell proliferation, apoptosis, and cell cycle assays

For 50% inhibitory concentration (IC_50_) determination and synergy matrixes, HMCLs were cultured in 96-well plates for 96 hours in the presence of increasing concentrations of the drugs. Cell number was determined using adenosine triphosphate quantification with CellTiter-Glo, according to manufacturer’s instructions (G7573, Promega).

For apoptosis and cell cycle assays, cells were cultured for 48 hours in the presence of the drugs. Q-VD-OPh (551476; Sigma Aldrich) was added 30 minutes before drug treatment. Apoptosis was measured using Annexin V and 7-Aminoactinomycin D (7-AAD) labeling by flow cytometry, according to manufacturer’s instructions (556421; BD biosciences). Cell cycle was determined according to manufacturer’s instructions (557892; BD Biosciences), using BrdU (5-bromo-2′-deoxyuridine) incorporation (1.5 hours) and labeling with anti-BrdU to identify S-phase cells, and DAPI (4′,6-diamidino-2-phenylindole) staining for total DNA.

Supplemental methods, reagents, and antibodies are included in supplemental Data.

## Results

### TRC-resolution genes are overexpressed in MM and associated with poor outcome

**Figure 1.**
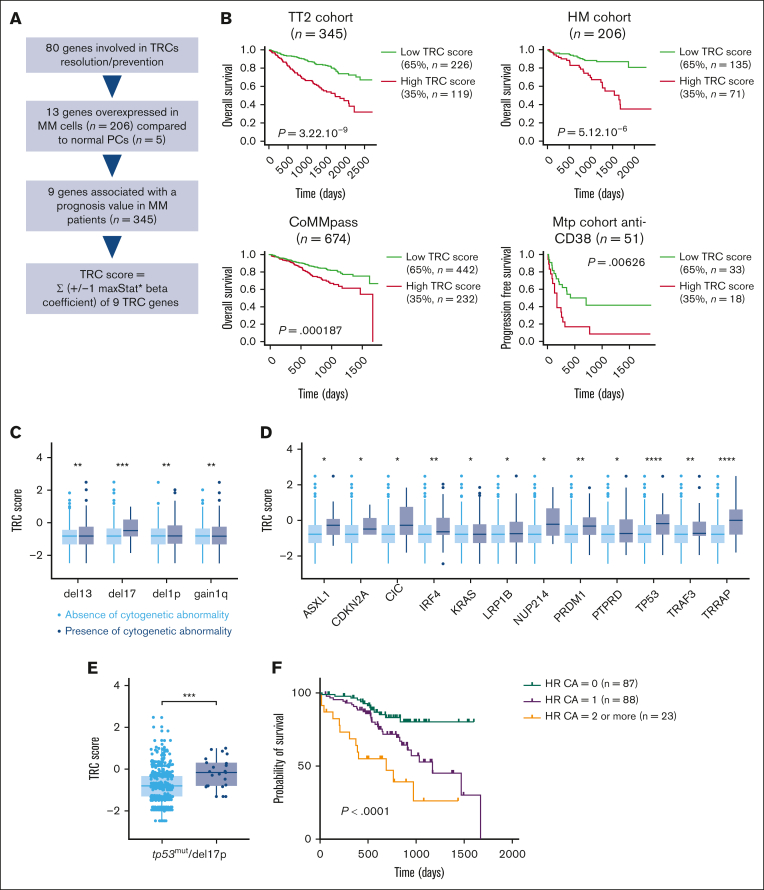
**TRC gene resolution–based score identifies high-risk patients with MM.** (A) Chart explains how the TRC score was built. (B) Prognostic value of the TRC score in newly diagnosed patients treated with high-dose chemotherapy and ASCT. Patients of the TT2 cohort (UAMS, n = 345) were ranked according to increased TRC score and a maximum difference was obtained with TRC score splitting patients into high-risk (n = 119) and low-risk (n = 226) groups. TRC score also had a prognostic value in 2 independent cohorts of newly diagnosed patients with MM treated with HDT and ASCT (HM, n = 206; CoMMpass, n = 674) and in a cohort of patients at relapse treated with anti-CD38 mAb (daratumumab; Montpellier [Mtp] cohort, n = 51). The parameters to compute the TRC score of patients in the HM, CoMMpass, and Mtp cohorts were those defined with the TT2 cohort. (C) TRC score in patients from the CoMMpass cohort without CA (light blue) and patients with CA (dark blue). (D) TRC score in patients from the CoMMpass cohort without the mutation (light blue) or with the mutation (dark blue) on the indicated gene. (E) TRC score in patients from the CoMMpass cohort with the double hit (dark blue) or without (light blue) *TP53*^mut^/del17p. (F) Kaplan-Meier overall survival of newly diagnosed patients with MM (CoMMpass cohort) with high TRC score according to the association with other high-risk CAs (HR CAs). HR CAs are defined by the presence of del(17p), t(4;14), and/or gain(1q). The green curve corresponds to patients with high TRC score without other HR CAs; the violet curve to patients with ≥1 CA; and the orange curve to patients with ≥2 other HR CAs. *P* value is determined by the log-rank test comparison.

We previously showed that 13 genes involved in TRC resolution were overexpressed in malignant PCs (n = 206) compared with normal PCs (n = 5), and high expression of 9 of these 13 genes predicted shorter overall survival in newly diagnosed patients with MM.^19^ Here, we used the prognostic value of these 9 genes (*BRIP1*, *DDX1*, *DDX23*, *EXOSC5*, *FANCD2*, *HNRNPU*, *PRMT5*, *SRPK2*, and *XRN2*) to build a gene expression profile–based score, called TRC score (Figure 1A; supplemental Table 1).^18^^,^^20^ The TRC score is defined by the sum of the beta coefficients from the Cox model for each prognostic gene, weighted by ±1 according to the patient MMC signal above or below the cut point expression value defined using maxstat R function, as previously described.^21^^,^^22^ High TRC score was associated with poor outcome in 4 independent cohorts of newly diagnosed patients with MM (UAMS, TT2 cohort, n = 345; HM, n = 206; CoMMpass, n = 674; Hovon, n = 282) treated with HDT and ASCT (Figure 1B; supplemental Figure 1A-B) and also in a cohort of patients at relapse treated with the anti-CD38 mAb daratumumab (Figure 1B). Importantly, the TRC score had no significant prognostic value in a cohort of cytogenetically normal acute myeloid leukemia (AML; n = 162), a cohort of chronic lymphocytic leukemia (Herold CLL cohort, n = 107), and a cohort of diffuse large B-cell lymphoma (Lenz-DLBCL rituximab, cyclophosphamide, doxorubicin, vincristine, and prednisone [R-CHOP] cohort, n = 233; supplemental Figure 1C). To validate the pathophysiological relevance of some genes in the TRC score, we investigated the role of DDX1 and DDX23 RNA helicases in MMCs. These helicases have been linked to TRC prevention through R-loops resolution.^23^^,^^24^ Depletion of *DDX1* and *DDX23* using inducible short hairpin RNAs decreased cell proliferation and induced apoptosis, together with phosphorylation of H2A.X, suggesting spontaneous DNA damage formation (supplemental Figure 2). Using a cohort of 112 patients with RNA-sequencing (RNA-seq) data from purified MMCs and the corresponding RNA-seq of the nontumor BM microenvironment, we analyzed the correlation between TRC score of purified MMCs and the abundance of immune cell subpopulations within the nontumor BM fraction.^25^ We estimated the abundance of the immune cell subpopulations within the RNA-seq data using the CIBERSORTx suite.^26^ Importantly, we identified a significant decrease in dendritic cell and macrophage BM abundance in patients with high TRC score compared with patients with low TRC score (supplemental Figure 3A), which may support the shorter progression-free survival of TRC score^high^ patients receiving anti-CD38 mAb therapy (Figure 1B). Furthermore, TRC score was significantly higher in patients with del(17)p, del13, del1p, 1q gain, *TP53*, *ASXL1*, *CDKN2A*, *IRF4*, *TRAF3*, or *TRRAP* mutations (Figure 1C-D). Transformation/Transcription Domain Associated Protein (TRRAP) is required for *MYC* and *TP53* transcription activation and plays a role in Double-strand break (DSB) repair.^27^^,^^28^ *TRRAP* mutation is associated with a poor outcome in patients with MM.^29^ *CDKN2A* encodes for p16 and p14, which are 2 tumor suppressor genes involved in cell cycle regulation and p53 activation.^30^ Interferon Regulatory Factor 4 (IRF4) is a transcription factor involved in a coregulatory loop with Myc in malignant PCs.^31^ TRAF3 is involved in tumor necrosis factor receptor signaling, regulates B-cell survival, and inhibits PC development through IL-6 receptor signaling inhibition.^32^, ^33^, ^34^ ASXL1 works with the Polycomb Repressive Complex 2 (PRC2) complex to repress gene expression, and *ASXL1* mutations result in increased expression of leukemogenic genes in myeloid malignancies.^35^^,^^36^ Moreover, the TRC score was significantly higher in patients with MM harboring double-hit del(17)p/*TP53* mutation, which are associated with worse prognosis and resistance to treatment (Figure 1E).^37^ In Cox analyses with high-risk cytogenetic abnormalities (CAs), the TRC score remained an independent prognostic factor compared with del17p or 1q gain (supplemental Figure 3B-D). As previously described, accumulating high-risk CAs worsen the prognosis of patients with MM.^38^, ^39^, ^40^, ^41^ We have assessed the effect of additional high-risk CAs (del17p, 1q gain, and t(4;14)) on the prognosis of patients with high-risk TRC score in the CoMMpass cohort. Not surprisingly, the OS of patients with high TRC score significantly decreased when this abnormality was associated with other high-risk CAs (*P* < .0001) (Figure 1F). Gene set enrichment analysis (GSEA) of genes upregulated in TRC^high^ patients identified significant enrichment in signatures related to cell cycle, proliferation, and plasmablastic stage (supplemental Figure 4A). However, the TRC score was not correlated with the PC labeling index (percentage of PCs in S-phase) in newly diagnosed patients with MM (n = 64; supplemental Figure 4B).^42^ These data indicate that a TRC-resolution machinery is significantly overexpressed in MM, together with proliferative plasmablastic signatures and high-risk molecular abnormalities, including del17p, 1q gain, *TP53* mutations, or double-hit genomic abnormalities (del17p/*TP53* mutation).^2^ This TRC-resolution machinery could therefore represent a therapeutic target for MMCs, by using new therapies such as G4 stabilizers.

### Increasing TRCs using G4 stabilizers affects MMC proliferation and survival

**Figure 2.**
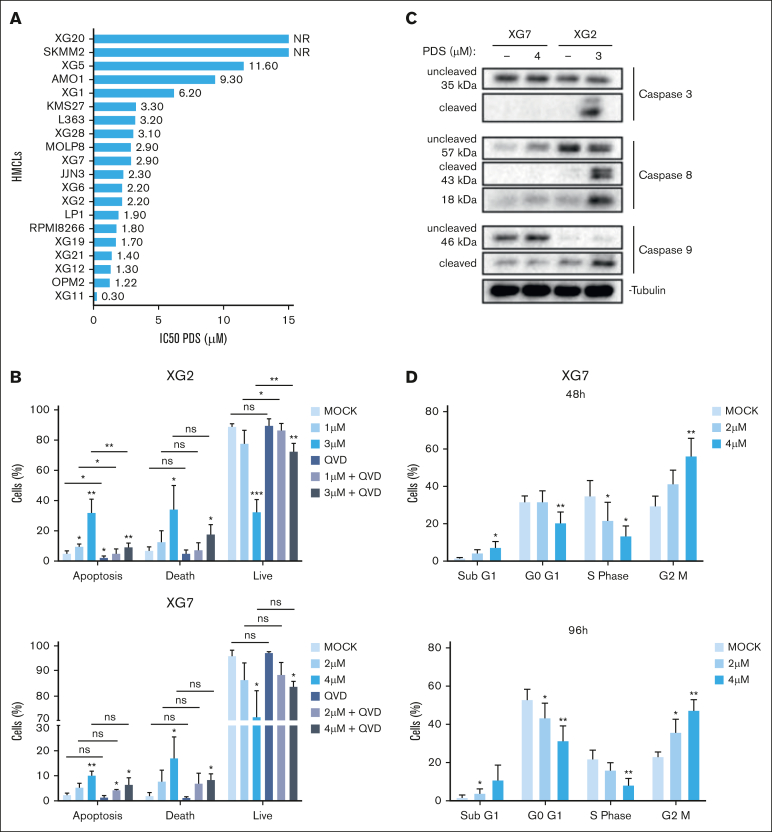
**G4s stabilization impairs HMCLs growth leading to cell death.** (A) Twenty HMCLs were cultured with increasing doses of PDS for 96 hours. Cell viability was determined using adenosine triphosphate (ATP) quantification. The IC_50_ of PDS is shown on top of the histograms. (B-C) XG2 and XG7 cells were cultured for 2 days with the pan-caspases inhibitor Q-VD-OPh (QVD; 20 μM) and PDS at the indicated doses. Apoptosis activation was analyzed using Annexin V and 7-AAD labeling by flow cytometry (B) and western blot detection of caspase cleavage (C). Apoptotic cells are Annexin V positive and 7-AAD negative, dead cells are 7-AAD positive and Annexin V positive, and live cells are double negative. Caspase 3, 8, and 9 cleavages were analyzed by western blot. One representative experiment of 3 is shown. Student *t* test, ∗*P* < .05; ∗∗*P* < .01; ∗∗∗*P* < .005. (D) XG7 cells were treated for 48 hours and 96 hours with the indicated concentrations of PDS. BrdU (10 µg/mL) was added during the last 1.5 hours of treatment. Cells were fixed and processed to detect BrdU incorporation and total DNA. BrdU^+^ cells were assigned to S phase. BrdU^–^ cells were assigned to G_0_/G_1_ or G_2_/M phases based on their DNA content. Student *t* test, ∗*P* < .05; ∗∗*P* < .01. Results are the mean of 3 independent experiments. NR, not reached; ns, not significant.

PDS is a highly specific G4 stabilizer that can increase TRCs.^43^ We tested the response to PDS in a large panel of HMCLs, representative of a large part of the molecular heterogeneity of patients with MM.^14^^,^^15^^,^^44^^,^^45^ Treatment with PDS was associated with significant toxicity with a median IC_50_ of 2.6 μM (Figure 2A). HMCL TRC score correlated with immunoglobulin secretion but not with their molecular subgroup (supplemental Figure 4C-D; supplemental Table 2). PDS induced a strong increase in apoptosis and cell death at 48 hours in 2 of 4 sensitive cell lines (XG2 and OPM2; but not XG21 or XG7), whereas the resistant cell line AMO1 did not show an increase in cell death upon PDS treatment (supplemental Figure 5A-B). Moreover, pan-caspase inhibition significantly rescued apoptosis and viability in XG2. In XG7, a decrease in apoptosis and cell death induction was identified, although not significant. This indicates that PDS induces caspase-dependent cell death in XG2 and to a lesser extent in XG7 cells, which could be explained by differential immunoglobulin secretion or unfolded protein response activation by PDS (Figure 2B; supplemental Figure 5C).^17^ Accordingly, XG2 but not XG7 cells showed cleavage of caspases 3, 8, and 9 after PDS treatment, as well as increased expression of proapoptotic Bcl-2 Interacting Mediator of cell death (BIM) and Bcl-2 homologous antagonist/killer (BAK) and antiapoptotic Bcl-2 (Figure 2C; supplemental Figure 5D). Furthermore, PDS induced an accumulation of cells in the G_2_/M phase, although to a lesser extent in the 2 resistant cell lines. It was correlated with a decrease in cells in S-phase for the 3 sensitive cell lines and a decrease in G_0_/G_1_ in XG7 cells (Figure 2D; supplemental Figure 6A). We also detected Poly (ADP-ribose) polymerase (PARP) cleavage, increased expression of p21, and downregulation of p27 (supplemental Figure 6B). We conclude that stabilization of G4s using PDS blocks the progression of cell cycle and can eventually lead to MMC death, as previously shown on other cancer cell lines.^15^

**Figure 3.**
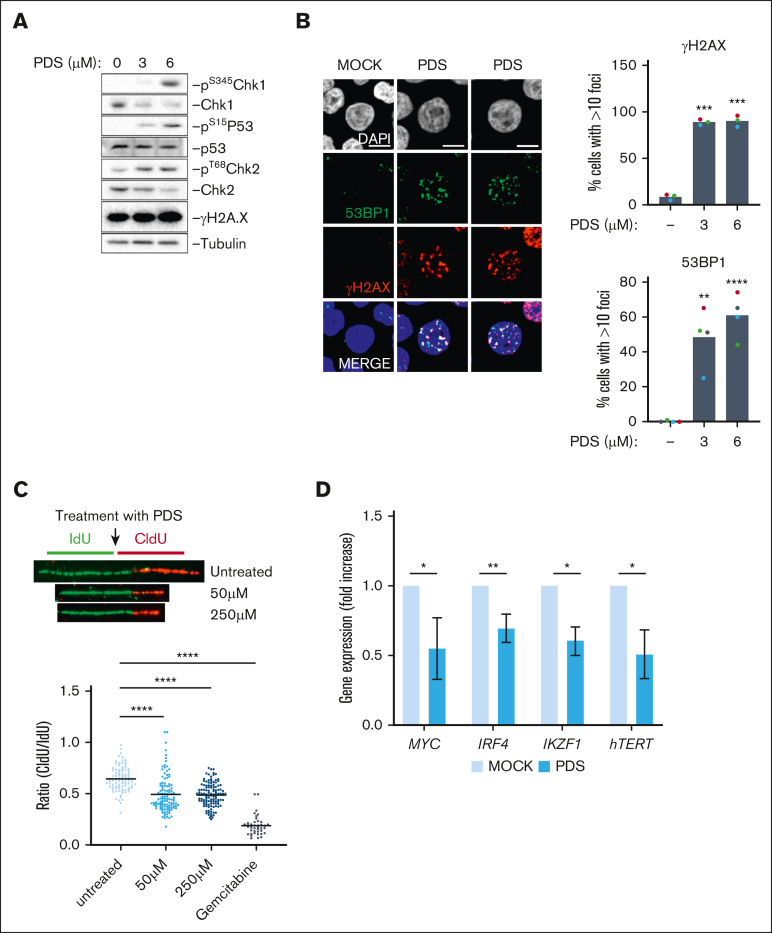
**G4s stabilization induces DNA damage and impairs replication fork progression in HMCLs.** (A) XG7 cells were treated for 24 hours with PDS at the indicated doses and were harvested for western blot detection of DDR activation. Images are representative of 3 independent experiments. (B) XG7 cells were treated for 24 hours with PDS at the indicated doses, collected, and used for immunofluorescence detection of γH2A.X and 53BP1 nuclear foci. Quantification of foci number per nucleus was done using ImageJ software. Results are the mean of 3 or 4 independent experiments. Student *t* test, ∗∗*P* < .01; ∗∗∗*P* < .005; ∗∗∗∗*P* < .0005. (C) XG7 cells were labeled for 30 minutes with IdU and then 30 minutes with CldU and with PDS (as indicated) or gemcitabine (10 μM). Cells were then collected for DNA fiber analysis. The lengths of the 5-iodo-2'-deoxyuridine (IdU) and 5-Chloro-2'-deoxyuridine (CldU) tracks were plotted as the ratio of CldU to IdU. Student *t* test, ∗∗∗∗*P* < .0005. (D) XG7 cells were treated with 1.25μM PDS for 48 hours and then analyzed by quantitative reverse transcription polymerase chain reaction. Results are the mean of 3 or 4 independent experiments. Student *t* test, ∗*P* < .05; ∗∗*P* < .01.

PDS has been shown to prevent replication fork progression and induce DNA damage.^14^ Therefore, the G_2_/M cell cycle arrest induced by PDS could be explained by DNA damage formation. Accordingly, PDS induced a wide DDR as shown by the phosphorylation of p53, Chk1, Chk2, and H2A.X (Figure 3A). This was associated with a significant increase in 53BP1 and γH2A.X foci formation and phosphorylation of RPA32 (Figure 3B; supplemental Figure 7A). Importantly, a short treatment with PDS significantly decreased replication fork progression and 5-Ethynyl-2'-deoxyuridine (EdU) incorporation (Figure 3C; supplemental Figure 7B). Additionally, a 48-hour PDS treatment led to a 50% decrease in the messenger RNA (mRNA) production of major MM oncogenes, namely *MYC* and *IRF4*, together with *IKZF1* transcription factor regulating IRF4 and Myc expression (Figure 3D).^31^ We also detected the downregulation of *hTERT*, a Myc target known to have G4s in its promoter.^46^ G4s can also form on RNA, and PDS has been shown to decrease ribosomal protein mRNA translation.^47^ We used polysome profiling to identify translation alterations induced by PDS in MMCs, but no significant changes were detected^48^ (supplemental Figure 7C). These results show that PDS can hinder MMC proliferation, which may be linked with the formation of DNA damage and the downregulation of major MM oncogenes.

**Figure 4.**
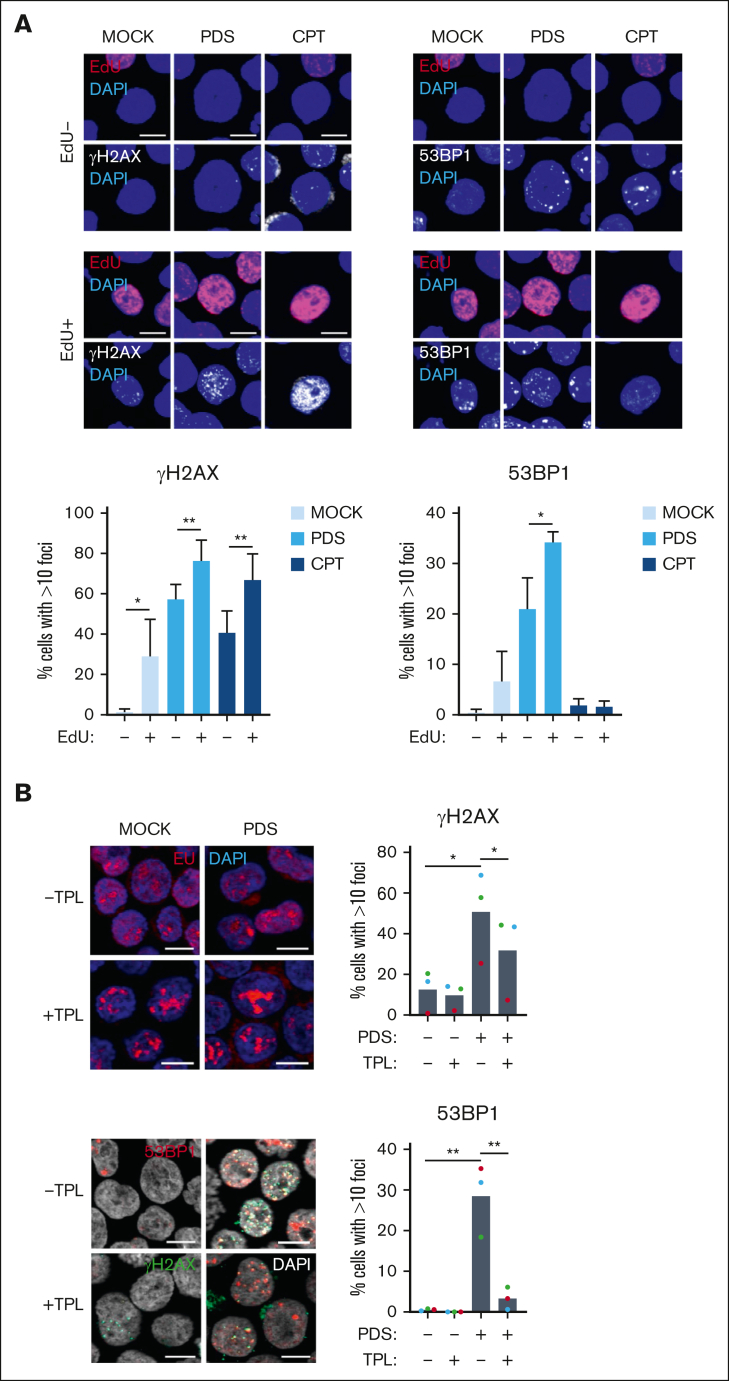
**Ongoing replication and active transcription are required for PDS-induced replication stress.** (A) XG7 cells were pulse-labeled with EdU followed by treatment with PDS (10 μM) and camptothecin (CPT; 1 μM) for 5 hours. γH2A.X and 53BP1 foci were detected using immunofluorescence and quantified in non–S-phase cells (EdU^–^) and in S-phase cells (EdU^+^) using Image J software. Results are the means of 3 independent experiments. Student *t* test, ∗*P* < .05; ∗∗*P* < .01. (B) XG7 cells were treated with triptolide (TPL; 10 μM) for 1 hour, followed by PDS treatment (1 hour; 10 μM). Results are the means of 3 independent experiments. Student *t* test, ∗*P* < .05; ∗∗*P* < .01.

To decipher the role of TRCs in the mechanism of action of PDS, we investigated the contribution of replication and transcription to the PDS-induced DNA damage. In untreated cells, 53BP1 and γH2A.X nuclear foci were detected only in S-phase (EdU^+^) cells, consistent with a replication-dependent increase of DNA damage during S phase and formation of constitutive DNA repair foci in MMCs (Figure 4A).^6^^,^^49^ Upon PDS exposure, 53BP1 and γH2A.X foci were detected in nonreplicating (EdU^–^) cells and were further increased in S-phase cells, indicating either that PDS can target both replicating and nonreplicating cells or that DNA lesions formed during S phase are transmitted to daughter cells in the following cell cycle. Pretreatment with the transcription inhibitor triptolide, which prevents TRCs by evicting RNA polymerase II (RNAPII) from chromatin,^50^^,^^51^ prevented the formation of 53BP1 foci in PDS-treated cells (Figure 4B). The reduction was only partial for γH2A.X foci, suggesting that transcription inhibition does not fully suppress RS, even though it prevents the formation of DSBs. To ensure that this result was not related to a decreased replication rate due to transcription inhibition, we used another transcription inhibitor, 5,6-dichloro-1-beta-D-ribofuranosylbenzimidazole (DRB), which stalls the RNAPII on the chromatin and therefore may increase TRCs.^50^ Interestingly, we found that DRB pretreatment did not significantly reduced the formation of γH2A.X and 53BP1 foci in PDS-treated cells, which may be explained by the RNAPII retention on the chromatin, promoting PDS-induced DNA damage (supplemental Figure 7D). Altogether, these data suggest that active transcription is necessary for PDS-induced DSBs in replicating cells, supporting the fact that PDS increases TRCs.

### G4 stabilization improve the efficacy of conventional MM treatments

**Figure 5.**
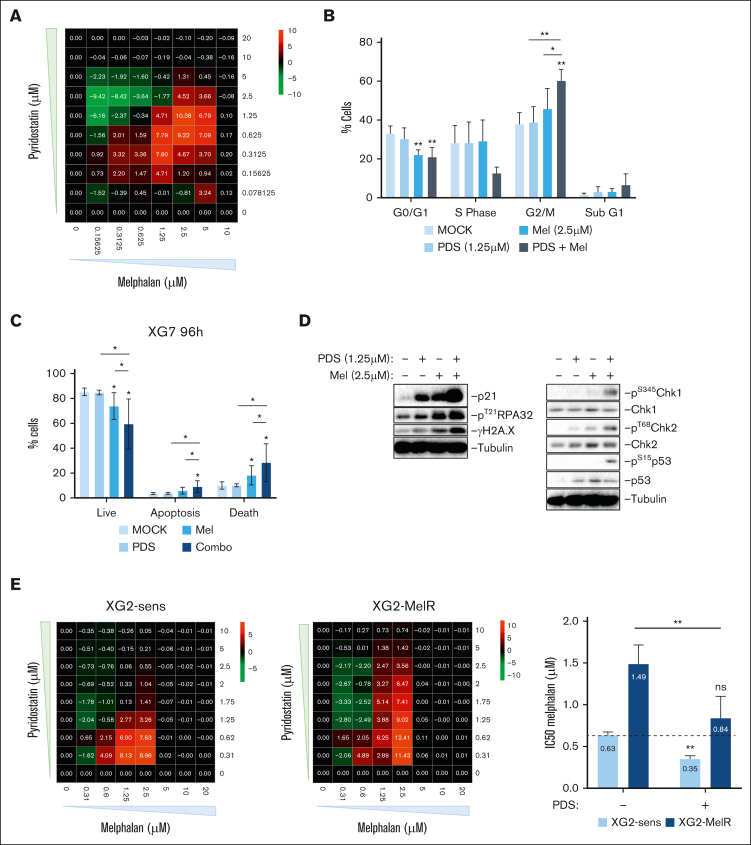
**G4s stabilization increases the efficacy of melphalan in HMCLs.** (A) XG7 cells were treated with increasing concentrations of PDS and melphalan for 96 hours. Cell viability was measured using ATP quantification to obtain a viability matrix. Positive (red) values indicate synergism and negative (green) values antagonism. (B) XG7 cells were treated for 48 hours with PDS and melphalan and were harvested for cell cycle distribution using BrdU incorporation and DAPI staining. Student *t* test, ∗*P* < .05; ∗∗*P* < .01. (C) XG7 cells were cultured for 96 hours with PDS (1.25 μM) and melphalan (2.5 μM). Apoptosis activation was analyzed using Annexin V and 7-AAD labeling by flow cytometry. Apoptotic cells are Annexin V positive and 7-AAD negative; dead cells are 7-AAD positive and Annexin V positive; and live cells are double negative. Student *t* test, ∗*P* < .05. (D) XG7 cells were treated for 48 hours with PDS and melphalan and were harvested for western blot analysis of the DDR. Images are representative of 3 independent experiments. (E) XG2-sens and XG2-melphalan–resistant (XG2-MelR) cell lines were treated with increasing concentrations of PDS and melphalan for 4 days and analyzed similar to panel A. Histogram (right) showing the IC_50_ of melphalan alone or supplemented with 1.25 μM of PDS in XG2-sens and XG2-MelR. Results are the mean of 4 independent experiments. Student *t* test, ∗∗*P* < .01. ns, not significant.

Because monotherapy is not effective in MM, we sought to identify potent combinations of PDS and currently used MM treatments based on a biological rationale. According to our previous results, we hypothesized that the RS induced by PDS could enhance the response to the alkylating agent melphalan. Using synergy matrixes, we identified a significant synergy when PDS was combined with melphalan in XG7, together with cell cycle arrest in G_2_/M and apoptosis (Figure 5A-C; supplemental Figure 8A). It also correlated with an increased activation of DDR compared with either drug alone (Figure 5D). We validated this synergy using XG2 and XG1 HMCLs (supplemental Figure 8B-D). These data show that targeting TRCs with PDS increases melphalan efficacy on HMCLs. Because melphalan resistance can emerge in patients and usually leads to relapse, the ability of PDS to improve the response of HMCLs to melphalan may represent a new strategy to overcome this resistance. We found a comparable synergistic profile between an XG2-melphalan–resistant cell line and its normal counterpart (XG2-sensitive [XG2-sens]; Figure 5E).^52^ The IC_50_ of melphalan in the presence of PDS on the resistant cell line was comparable with the IC_50_ of melphalan alone on the parental sensitive cell line (Figure 5E). PDS and melphalan led to increased γH2A.X and pRPA32 in both XG2-sens and XG2-melphalan–resistant, as well as an increased phosphorylation of Chk1 but not of p53 compared with XG2-sens (supplemental Figure 8E). pChk2 was not increased in any cell line. This indicates that PDS can increase the DNA damage induced by melphalan and may be of interest to overcome melphalan resistance in MM.

We showed that PDS led to a decreased production of *IKZF1*, *IRF4*, and *MYC* mRNAs (Figure 3D). Immunomodulatory drugs (IMiDs) target the degradation of IKZF1 and IKZF3 through binding to cereblon, thereby inhibiting *IRF4* and *MYC* transcription.^53^ Thus, we reasoned that combining PDS and IMiDs would increase the targeting of the Myc-IRF4 regulatory loop. We found that the combination of PDS with either lenalidomide or pomalidomide was associated with a strong synergy in XG7 and JJN3 but not in XG20, XG2, or L363 (supplemental Figure 9A-B). It was associated with decreased protein expression of Ikaros, Myc, and IRF4 (supplemental Figure 10A). GSEA of the dysregulated genes upon cotreatment with lenalidomide and PDS revealed a significant induction of BMP2 targets, KDM1A/LSD1 targets, B-cell signatures, immune response, and upregulation of genes downregulated in patients with MM overexpressing *MYC* and *BCL2L1* (supplemental Figure 10B). Of interest, BMP2 was shown to induce MMC apoptosis.^54^^,^^55^ Furthermore, mutations of *LSD1*/*KDM1A* was shown to confer susceptibility to MM.^56^ These data indicate that the combination of G4 stabilizers with IMiDs could be an interesting strategy to target MMCs. Proteasome inhibitors are also used for the treatment of MM and have greatly improved the survival of patients. These drugs have been shown to inhibit the DDR,^57^^,^^58^ and combining PDS with bortezomib or carfilzomib was associated with a modest synergy in XG7 and no synergy in XG2 and XG1 (supplemental Figure 10C-D). Importantly, we found that the synergy between PDS and lenalidomide was not observed on AML and MCL cell lines but was conserved on a DLBCL cell line (supplemental Figure 11A). The combination of PDS and bortezomib was associated with modest synergy on an AML and a DLBCL cell line but not on an MCL cell line (supplemental Figure 11B). Taken together, these data show that G4 stabilizers could be combined with current MM therapies such as alkylating agents or IMiDs, based on biological rationale involving the main effects of G4 ligands, such as TRC-mediated DNA damage or oncogene downregulation.

### G4 stabilization and chromatin acetylation increases MMC death

**Figure 6.**
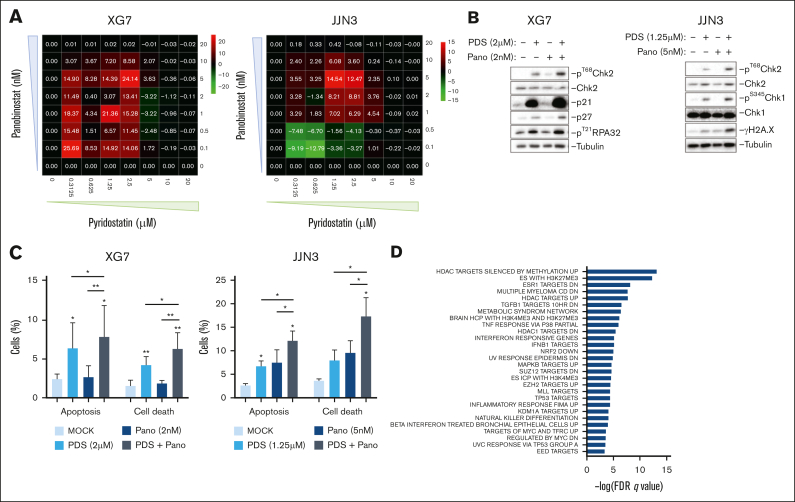
**G4s stabilization increases the efficacy of panobinostat in HMCLs.** (A) XG7 and JJN3 were treated with increasing concentrations of PDS combined with panobinostat for 96 hours. Positive (red) values indicate synergism and negative (green) values antagonism. (B) XG7 and JJN3 cells were treated for 48 hours with PDS and panobinostat at the indicated doses and harvested for western blot. Images are representative of 3 independent experiments. (C) XG7 and JJN3 cells were treated for 48 hours with PDS and panobinostat at the indicated doses and harvested for apoptosis analysis using Annexin V and 7-AAD labeling. Apoptotic cells are Annexin V positive and 7-AAD negative; and dead cells are Annexin V positive and 7-AAD positive. Student *t* test, ∗*P* < .05; ∗∗*P* < .01. (D) XG7, JJN3, and XG2 cells were treated for 48 hours with PDS, panobinostat, or the combination of both drugs, and mRNA was sequenced. GSEA of upregulated genes in cells treated with PDS and panobinostat compared with control cells is represented. Differential expression was assessed using DESeq2 R package (log[fold change] > 1.5; *P* < .05). FDR, false discovery rate.

Histone acetylation can increase fork velocity and fork stalling in an R-loop–dependent manner.^59^ Interestingly, we identified that HMCLs and primary MM samples with a high TRC score were significantly more sensitive to the histone deacetylase (HDAC) inhibitor (HDACi) panobinostat, currently used for the treatment of patients with MM at relapse (supplemental Figure 12A-B). We reasoned that increasing R-loop formation with panobinostat while stabilizing these R-loops with PDS should have a synergistic effect. Accordingly, the combination of PDS and panobinostat had a synergistic effect in 3 HMCLs (XG7, JJN3, and XG2; Figure 6A; supplemental Figure 12C). The combination increased the phosphorylation of Chk1 and Chk2 in both XG7 and JJN3 cells, along with increased phosphorylation of RPA32 in XG7 cells and of H2A.X in JJN3 cells (Figure 6B). It was also associated with increased apoptosis and cell death in both cell lines (Figure 6C). In addition, a significant accumulation of cells in G_2_/M was detected for JJN3 but not for XG7 cells (supplemental Figure 12D-E). In line with a study using the G4 ligand CX-5461, the synergy between PDS and panobinostat was also identified in p53-depleted cells (supplemental Figure 12F), showing that this effect was not dependent on p53 status.^60^ To gain insight into the pathways deregulated by the drug combination, we performed RNA-seq analysis of XG7, XG2, and JJN3 cells after 48 hours of treatment with PDS and panobinostat. Differential expression analysis for sequence count data 2 (DESeq2) analysis of PDS-treated cells showed that 15 genes were deregulated with PDS, without association with any gene signature (supplemental Figure 13A). GSEA of panobinostat-treated cells confirmed the upregulation of HDAC targets and identified other epigenetic signatures related to Polycomb group protein activity. Moreover, a signature related to innate immune system activation was upregulated, as well as pathways linked with p53 and MAPK8 activity (supplemental Figure 13B). In the combo-treated cells, we found the same enrichment identified in panobinostat-treated cells, namely HDAC and Polycomb targets, and immune system activation but with higher false discovery rate (FDR) q values (Figure 6D). Moreover, alterations in metabolism, cell signaling, and Myc transcriptional activities were identified. These data indicate that the combination of PDS and panobinostat results in increased DNA damage and DDR activation, leading to cell cycle arrest and cell death, possibly along with epigenetic, signaling, and metabolism deregulations.

BRDis bind to bromodomain and extraterminal (BET) domains and impair BET protein recognition of acetylated chromatin, preventing transcriptional pause release.^7^ Importantly, these molecules can increase R-loop formation as well as inhibiting *MYC* expression in MMCs.^50^^,^^61^^,^^62^ We found a significant correlation between high TRC score value and sensitivity to 2 BRDis in HMCLs (supplemental Figure 14A-B). Furthermore, PDS combination with I-BET-762 demonstrated synergistic activity in HMCLs (supplemental Figure 14C-D). The combination was associated with a decreased protein expression of Myc, Ikaros, and IRF4 (supplemental Figure 14E). BRDis were shown to downregulate genes regulated by superenhancers (SEs), and G4s could also be present in SEs.^63^ Interestingly, *IRF4* and *MYC* genes were enriched with large peaks of H3K27ac and H3K4me1 histones marks typical of SEs (supplemental Figure 15A). Moreover, γH2A.X was strongly increased, and the activation of p21 by PDS was diminished with the addition of BRDi (supplemental Figure 14E). Altogether, these data suggest that combining PDS and BRDi enhances the targeting of the IRF4-Myc loop and increases TRC-mediated and R-loop–mediated DNA damage, resulting in decreased proliferation, as well as a possible defect in DNA damage checkpoint activation.

### PDS kills primary MMCs and synergizes with HDACi and BRDi

**Figure 7.**
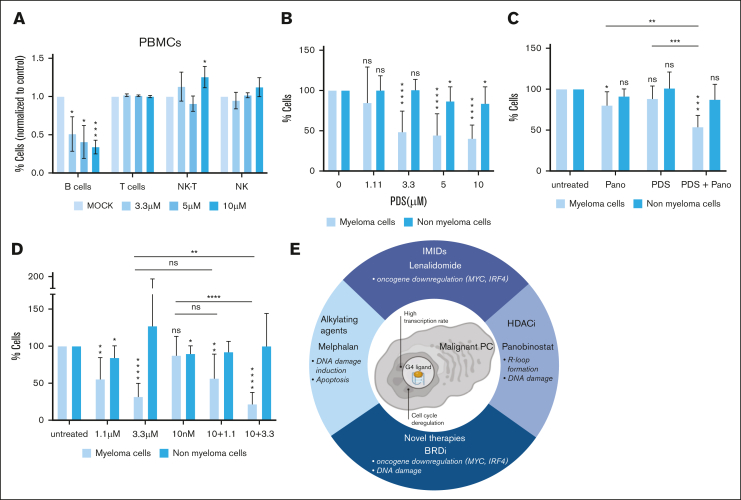
**PDS increases the efficacy of MM treatments on primary MMCs.** (A) Peripheral blood mononuclear cells (PBMCs) from 4 healthy donors were cultured for 4 days with increasing doses of PDS. B cells, T cells, NK cells, and NK-T cells were identified using CD45, CD19, CD3, and CD56 labeling by flow cytometry. (B) Mononuclear cells from tumor samples of 15 patients with MM were cultured for 4 days in the presence of IL-6 (2 ng/mL) with or without increasing concentrations of PDS. At day 4 of culture, the count of viable MMCs was determined using CD138 and CD38 staining by flow cytometry. (C) Primary cells of 6 patients with MM were cocultured with the normal cells from the BM microenvironment and treated with PDS (1.1 μM) and panobinostat (Pano; (2.5 nM). Cytotoxicity was determined as described in panel A. (D) Primary cells of 5 patients with MM were cocultured with the normal cells from the BM microenvironment and treated with PDS (1.1 μM and 3.3 μM) and I-BET-762 (BRDi, 10 nM). Cytotoxicity was determined as described in panel A. Student *t* test, ∗*P* < .05; ∗∗*P* < .01; ∗∗∗*P* < .005; ∗∗∗∗*P* < .0005. (E) Chart representing the possible therapeutic combinations involving PDS and their respective consequences on MMCs. ns, not significant.

To validate the therapeutic potential of G4 stabilization in MM, we first investigated the toxicity of PDS on normal peripheral blood mononuclear cells from healthy donor (n = 4). We found significant toxicity on B cells without toxicity on T cells, natural killer cells (NK), and NK-T cells (Figure 7A). Cell count and viability of peripheral blood mononuclear cells were not significantly affected by PDS (supplemental Figure 16A-B). We then investigated the toxicity of PDS on normal BM cells as well as primary MMCs. BM samples of patients with MM were cultured in the presence of IL-6 and increasing doses of PDS, and specific toxicity on PCs and BM cells was investigated with CD38 and CD138 detection. We found that PDS could target primary MMCs in a dose-dependent manner and had a significant but slight effect on normal BM cells (Figure 7B). The combination of PDS and panobinostat or I-BET-762 synergistically targeted primary MMCs from patients without significant toxicity on BM cells or CD34^+^ progenitor cells (Figure 7C-D; supplemental Figure 16C). These combinations resulted in increased γH2A.X staining in CD138^+^ cells (supplemental Figure 16D). Together, these data indicate that the combination of PDS with panobinostat or BRDi specifically targets MMCs over normal BM cells.

## Discussion

RS is a major source of genomic instability in MM. Highly transcribed regions in normal PCs are involved in *IGH* translocations and mutations in malignant PCs.^19^^,^^64^ We, therefore, reasoned that malignant PCs could be particularly sensitive to TRCs, a feature that could be exploited for therapy. Consistent with this, we observed that increasing TRCs with a G4-stabilizing drug exacerbated RS to unbearable levels in HMCLs and primary MMCs. Importantly, G4 ligands also increased the efficacy of current MM treatments, such as melphalan, HDACi, proteasome inhibitors, or IMiDs, as well as BRDis currently in clinical development (Figure 7E).

Based on the expression of 9 TRC-resolution genes that we have previously reported to be upregulated in malignant PCs compared with normal bone marrow plasma cells (BMPCs), we designed a TRC score predictive of the survival of patients with MM in 4 independent cohorts of patients with MM.^19^ A high TRC score was associated with *TP53* alterations and mutations affecting cell cycle, DSB repair, or p53-related genes. Accordingly, TRC^high^ patients were associated with a downregulation of EZH2 targets and strong proliferation, plasmablastic, and cell cycle activation signatures. These findings suggest that the TRC score could be used to identify at diagnosis patients who could benefit from a TRC-enhancing therapy. Furthermore, the identification of lower immune cell abundance in TRC^high^ BM samples could explain the poor prognosis of patients after anti-CD38 therapy. RS, DNA damage, and, recently, p53 have been involved in the activation of cyclic GMP-AMP Synthase-stimulator of interferon genes (cGAS-STING) leading to a type I interferon response that could stimulate immune cells and antitumor immunity.^65^, ^66^, ^67^ Accordingly, we found that increasing the replication stress (RS) with PDS led to an increased expression of interferon-sensitive genes (supplemental Figure 15B). The overexpression of a TRC-managing machinery may, therefore, decrease the cellular response to DNA damage and the secretion of proinflammatory cytokines through the cGAS-STING pathway, explaining the lower abundance of immune cells.

G4 stabilization with PDS targeted HMCLs and primary MMCs, causing growth arrest and DNA damage. Very recently, a G4-specific photosensitizer was shown to trigger ROS generation and DNA damage and showed good efficacy to target malignant cells compared with normal cells, demonstrating the growing interest of G4 targeting to cure cancers.^68^ Two G4 stabilizers, CX-3543 and CX-5461, entered clinical trials for advanced hematological malignancies, including MM, though designated as RNA Pol I inhibitors and were withdrawn due to lack of efficacy. Yet, 10 years ago, the DNA damage induced by G4 stabilizers was shown to be repaired through homologous recombination, thus redirecting CX-5461 clinical trials for homologous recombination–mutated tumors, in which it showed better efficacy, good tolerance, and dose-limiting phototoxicity as the major adverse effect.^69^, ^70^, ^71^ No clinical trial was conducted using PDS. However, PDS treatment was associated with memory deficiency in aged mice, indicating that this compound might have neurotoxicity in vivo.^72^^,^^73^ This side effect was not observed with CX-5461 G4 stabilizer in clinical trials.^70^^,^^71^ This demonstrates that G4 stabilizers could be particularly interesting in combination with drugs targeting DNA damage and RS. Specifically, because a high TRC score is associated with *TP53* and DNA damage–related genes alterations, it could be used to identify high-risk patients with MM who may benefit from a G4 stabilizer treatment to improve their response to conventional treatments. Accordingly, we found that PDS combination with the alkylating agent melphalan led to increased DNA damage, cell cycle arrest, and apoptosis and may overcome melphalan resistance. The toxicity of these combinations should be investigated in vivo.

To evaluate whether the TRC score could be used to predict treatment responses, we correlated HMCL TRC scores with their response to several drugs and identified a correlation between a high TRC score and increased sensitivity to panobinostat and 2 BRDis. Importantly, the correlation between TRC score and panobinostat response was validated using primary MMCs cocultured with BM microenvironment. Furthermore, we found synergies between PDS and these treatments on HMCLs and primary MMCs. This illustrates that the TRC score may be of interest for drug combinations involving G4 stabilizers.

Numerous studies have also reported the ability of G4 stabilizers to alter the expression of oncogenes with G4-associated promoters such as *c-MYC*. We confirmed the downregulation of *MYC* expression in HMCLs with PDS and identified a concomitant downregulation of *IRF4*. We confirmed that the synergy between PDS and panobinostat was conserved in p53-depleted cells. Furthermore, we identified a synergy between lenalidomide and PDS in a lenalidomide-resistant cell line but not in a sensitive one. This could mean that this synergy is only beneficial in an IMiD-resistance context, although we cannot exclude that a synergy could exist between PDS and pomalidomide or cereblon E3 ligase modulators (CELMoDs). Interestingly, PDS has been shown to modulate the immune system by inducing the interferon pathway in cancer cells. IMiDs stimulate the secretion of IL-2 by T cells, leading to the activation of NK cells. Moreover, by combining PDS and panobinostat, we identified an induction of the interferon pathway gene signature. It is, therefore, reasonable to suggest that the combination of PDS with IMiDs or HDACis could enhance immune system activation, a feature that would be particularly beneficial in the context of immune-based therapies.^74^ Very recently, a G4 and HDAC dual-targeting agent could target breast cancer cells, showing that the concomitant opening of the chromatin and stabilization of G4s is a promising strategy for cancer treatment.^75^ The action of IMiDs has been linked to the IRF4-MYC loop inhibition.^76^ However, IMiD resistance, which may involve reduced cereblon protein levels in MMCs, is associated with relapse.^53^ BET protein inhibition was shown to selectively decrease SE-associated genes, such as *MYC*, and entered clinical trials for patients with MM.^77^ We previously established an SE risk score based on the expression of 28 SE-associated genes linked with shorter overall survival in patients with MM.^45^ This score was associated with poor prognosis in MM and increased sensitivity to BRDi in HMCLs. Interestingly, the combination of PDS and BRDi resulted in a significant decrease in IRF4 and MYC expression, associated with SE in MMC lines, compared with PDS or BRDi alone. These results could explain the synergistic activity of this combination on MMCs. Moreover, we found increased DNA damage with the combination, which could be linked to increased collisions between RNA Pol II and replication due to R-loop formation. Because BRD proteins are also involved in the regulation of topoisomerase I activity, this DNA damage could be linked to increased topoisomerase I blocking.^50^^,^^61^

In conclusion, TRC targeting through G4 stabilization could be used to enhance the efficacy of current myeloma treatments, such as melphalan, HDACi, or BRDi, and thus provide a new strategy to improve outcomes of patients with MM.

Conflict-of-interest disclosure: The authors declare no competing financial interests.

## References

[1] Kumar SK, Rajkumar V, Kyle RA (2017). Multiple myeloma. Nat Rev Dis Primers.

[2] Corre J, Perrot A, Caillot D (2021). del(17p) without TP53 mutation confers a poor prognosis in intensively treated newly diagnosed patients with multiple myeloma. Blood.

[3] van de Donk NWCJ, Pawlyn C, Yong KL (2021). Multiple myeloma. Lancet.

[4] Xu L, Wen C, Xia J, Zhang H, Liang Y, Xu X (2024). Targeted immunotherapy: harnessing the immune system to battle multiple myeloma. Cell Death Discov.

[5] Kotsantis P, Petermann E, Boulton SJ (2018). Mechanisms of oncogene-induced replication stress: jigsaw falling into place. Cancer Discov.

[6] Walters DK, Wu X, Tschumper RC (2011). Evidence for ongoing DNA damage in multiple myeloma cells as revealed by constitutive phosphorylation of H2AX. Leukemia.

[7] Bowry A, Kelly RDW, Petermann E (2021). Hypertranscription and replication stress in cancer. Trends Cancer.

[8] Kotsantis P, Silva LM, Irmscher S (2016). Increased global transcription activity as a mechanism of replication stress in cancer. Nat Commun.

[9] Hoang PH, Cornish AJ, Dobbins SE, Kaiser M, Houlston RS (2019). Mutational processes contributing to the development of multiple myeloma. Blood Cancer J.

[10] Miglietta G, Russo M, Capranico G (2020). G-quadruplex–R-loop interactions and the mechanism of anticancer G-quadruplex binders. Nucleic Acids Res.

[11] Bochman ML, Paeschke K, Zakian VA (2012). DNA secondary structures: stability and function of G-quadruplex structures. Nat Rev Genet.

[12] Kosiol N, Juranek S, Brossart P, Heine A, Paeschke K (2021). G-quadruplexes: a promising target for cancer therapy. Mol Cancer.

[13] Carvalho J, Mergny J-L, Salgado GF, Queiroz JA, Cruz C (2020). G-quadruplex, friend or foe: the role of the G-quartet in anticancer strategies. Trends Mol Med.

[14] Rodriguez R, Müller S, Yeoman JA, Trentesaux C, Riou J-F, Balasubramanian S (2008). A novel small molecule that alters shelterin integrity and triggers a DNA-damage response at telomeres. J Am Chem Soc.

[15] Rodriguez R, Miller KM, Forment JV (2012). Small molecule-induced DNA damage identifies alternative DNA structures in human genes. Nat Chem Biol.

[16] De Magis A, Manzo SG, Russo M (2019). DNA damage and genome instability by G-quadruplex ligands are mediated by R loops in human cancer cells. Proc Natl Acad Sci U S A.

[17] Moreaux J, Klein B, Bataille R (2011). A high-risk signature for patients with multiple myeloma established from the molecular classification of human myeloma cell lines. 1.

[18] Moreaux J, Rème T, Leonard W (2012). Development of gene expression-based score to predict sensitivity of multiple myeloma cells to DNA methylation inhibitors. Mol Cancer Ther.

[19] Dutrieux L, Lin Y-L, Lutzmann M (2021). Transcription/replication conflicts in tumorigenesis and their potential role as novel therapeutic targets in multiple myeloma. Cancers (Basel).

[20] Kassambara A, Hose D, Moreaux J (2012). Genes with a spike expression are clustered in chromosome (sub)bands and spike (sub)bands have a powerful prognostic value in patients with multiple myeloma. Haematologica.

[21] Herviou L, Kassambara A, Boireau S (2018). PRC2 targeting is a therapeutic strategy for EZ score defined high-risk multiple myeloma patients and overcome resistance to IMiDs. Clin Epigenetics.

[22] Devin J, Cañeque T, Lin Y-L (2022). Targeting cellular iron homeostasis with ironomycin in diffuse large B-cell lymphoma. Cancer Res.

[23] de Amorim JL, Leung SW, Haji-Seyed-Javadi R (2024). The putative RNA helicase DDX1 associates with the nuclear RNA exosome and modulates RNA/DNA hybrids (R-loops). J Biol Chem.

[24] Sridhara SC, Carvalho S, Grosso AR, Gallego-Paez LM, Carmo-Fonseca M, de Almeida SF (2017). Transcription dynamics prevent RNA-mediated genomic instability through SRPK2-dependent DDX23 phosphorylation. Cell Rep.

[25] Bruyer A, Dutrieux L, de Boussac H (2023). Combined inhibition of Wee1 and Chk1 as a therapeutic strategy in multiple myeloma. Front Oncol.

[26] Steen CB, Liu CL, Alizadeh AA, Newman AM (2020). Profiling cell type abundance and expression in bulk tissues with CIBERSORTx. Methods Mol Biol.

[27] Murr R, Vaissière T, Sawan C, Shukla V, Herceg Z (2007). Orchestration of chromatin-based processes: mind the TRRAP. Oncogene.

[28] Murr R, Loizou JI, Yang Y-G (2006). Histone acetylation by Trrap-Tip60 modulates loading of repair proteins and repair of DNA double-strand breaks. Nat Cell Biol.

[29] Alaterre E, Vikova V, Kassambara A (2021). RNA-sequencing-based transcriptomic score with prognostic and theranostic values in multiple myeloma. J Pers Med.

[30] Elnenaei MO, Gruszka-Westwood AM, A’Hernt R (2003). Gene abnormalities in multiple myeloma; the relevance of TP53, MDM2, and CDKN2A. Haematologica.

[31] Shaffer AL, Emre NCT, Lamy L (2008). IRF4 addiction in multiple myeloma. Nature.

[32] Yi Z, Lin WW, Stunz LL, Bishop GA (2014). Roles for TNF-receptor associated factor 3 (TRAF3) in lymphocyte functions. Cytokine Growth Factor Rev.

[33] Moore CR, Edwards SK, Xie P (2015). Targeting TRAF3 downstream signaling pathways in B cell neoplasms. J Cancer Sci Ther.

[34] Lin WW, Yi Z, Stunz LL, Maine CJ, Sherman LA, Bishop GA (2015). The adaptor protein TRAF3 inhibits interleukin-6 receptor signaling in B cells to limit plasma cell development. Sci Signal.

[35] Medina EA, Delma CR, Yang F-C (2022). ASXL1/2 mutations and myeloid malignancies. J Hematol Oncol.

[36] Abdel-Wahab O, Adli M, LaFave LM (2012). ASXL1 mutations promote myeloid transformation through loss of PRC2-mediated gene repression. Cancer Cell.

[37] Martello M, Poletti A, Borsi E (2022). Clonal and subclonal TP53 molecular impairment is associated with prognosis and progression in multiple myeloma. Blood Cancer J.

[38] Perrot A, Lauwers-Cances V, Tournay E (2019). Development and validation of a cytogenetic prognostic index predicting survival in multiple myeloma. J Clin Oncol.

[39] Walker BA, Boyle EM, Wardell CP (2015). Mutational spectrum, copy number changes, and outcome: results of a sequencing study of patients with newly diagnosed myeloma. J Clin Oncol.

[40] Baysal M, Demirci U, Umit E (2020). Concepts of double hit and triple hit disease in multiple myeloma, entity and prognostic significance. Sci Rep.

[41] Weinhold N, Salwender HJ, Cairns DA (2021). Chromosome 1q21 abnormalities refine outcome prediction in patients with multiple myeloma - a meta-analysis of 2,596 trial patients. Haematologica.

[42] Requirand G, Robert N, Boireau S (2019). BrdU incorporation in multiparameter flow cytometry: a new cell cycle assessment approach in multiple myeloma. Cytometry Part B Clinical.

[43] Liu L-Y, Ma T-Z, Zeng Y-L, Liu W, Mao Z-W (2022). Structural basis of pyridostatin and its derivatives specifically binding to G-quadruplexes. J Am Chem Soc.

[44] Vikova V, Jourdan M, Robert N (2019). Comprehensive characterization of the mutational landscape in multiple myeloma cell lines reveals potential drivers and pathways associated with tumor progression and drug resistance. Theranostics.

[45] Alaterre E, Ovejero S, Herviou L (2022). Comprehensive characterization of the epigenetic landscape in multiple myeloma. Theranostics.

[46] Monsen RC (2023). Higher-order G-quadruplexes in promoters are untapped drug targets. Front Chem.

[47] Varshney D, Cuesta SM, Herdy B, Abdullah UB, Tannahill D, Balasubramanian S (2021). RNA G-quadruplex structures control ribosomal protein production. Sci Rep.

[48] Chassé H, Boulben S, Costache V, Cormier P, Morales J (2017). Analysis of translation using polysome profiling. Nucleic Acids Res.

[49] Matthews HK, Bertoli C, De Bruin RAM (2022). Cell cycle control in cancer. Nat Rev Mol Cell Biol.

[50] Edwards DS, Maganti R, Tanksley JP (2020). BRD4 prevents R-loop formation and transcription-replication conflicts by ensuring efficient transcription elongation. Cell Rep.

[51] Heuzé J, Kemiha S, Barthe A (2023). RNase H2 degrades toxic RNA:DNA hybrids behind stalled forks to promote replication restart. EMBO J.

[52] Ovejero S, Viziteu E, Dutrieux L (2022). The BLM helicase is a new therapeutic target in multiple myeloma involved in replication stress survival and drug resistance. Front Immunol.

[53] Bird S, Pawlyn C (2023). IMiD resistance in multiple myeloma: current understanding of the underpinning biology and clinical impact. Blood.

[54] Seher A, Lagler C, Stühmer T (2017). Utilizing BMP-2 muteins for treatment of multiple myeloma. PLoS One.

[55] Kawamura C, Kizaki M, Ikeda Y (2002). Bone morphogenetic protein (BMP)-2 induces apoptosis in human myeloma cells. Leuk Lymphoma.

[56] Wei X, Calvo-Vidal MN, Chen S (2018). Germline lysine-specific demethylase 1 (LSD1/KDM1A) mutations confer susceptibility to multiple myeloma. Cancer Res.

[57] Jacquemont C, Taniguchi T (2007). Proteasome function is required for DNA damage response and Fanconi anemia pathway activation. Cancer Res.

[58] Neri P, Ren L, Gratton K (2011). Bortezomib-induced “BRCAness” sensitizes multiple myeloma cells to PARP inhibitors. Blood.

[59] Salas-Armenteros I, Pérez-Calero C, Bayona-Feliu A, Tumini E, Luna R, Aguilera A (2017). Human THO–Sin3A interaction reveals new mechanisms to prevent R-loops that cause genome instability. EMBO J.

[60] Lee HC, Wang H, Baladandayuthapani V (2017). RNA polymerase I inhibition with CX-5461 as a novel therapeutic strategy to target MYC in multiple myeloma. Br J Haematol.

[61] Lam FC, Kong YW, Huang Q (2020). BRD4 prevents the accumulation of R-loops and protects against transcription–replication collision events and DNA damage. Nat Commun.

[62] Agnarelli A, Mitchell S, Caalim G (2022). Dissecting the impact of bromodomain inhibitors on the interferon regulatory factor 4-MYC oncogenic axis in multiple myeloma. Hematol Oncol.

[63] Fang S, Liu S, Yang D, Yang L, Hu C-D, Wan J (2022). Decoding regulatory associations of G-quadruplex with epigenetic and transcriptomic functional components. Front Genet.

[64] Lohr JG, Stojanov P, Carter SL (2014). Widespread genetic heterogeneity in multiple myeloma: implications for targeted therapy. Cancer Cell.

[65] Hopfner K-P, Hornung V (2020). Molecular mechanisms and cellular functions of cGAS–STING signalling. Nat Rev Mol Cell Biol.

[66] Técher H, Pasero P (2021). The replication stress response on a narrow path between genomic instability and inflammation. Front Cell Dev Biol.

[67] Ghosh M, Saha S, Li J, Montrose DC, Martinez LA (2023). p53 engages the cGAS/STING cytosolic DNA sensing pathway for tumor suppression. Mol Cell.

[68] Deiana M, Andrés Castán JM, Josse P (2023). A new G-quadruplex-specific photosensitizer inducing genome instability in cancer cells by triggering oxidative DNA damage and impeding replication fork progression. Nucleic Acids Res.

[69] Jin M, Hurley LH, Xu H (2023). A synthetic lethal approach to drug targeting of G-quadruplexes based on CX-5461. Bioorg Med Chem Lett.

[70] Khot A, Brajanovski N, Cameron DP (2019). First-in-human RNA polymerase I transcription inhibitor CX-5461 in patients with advanced hematologic cancers: results of a phase I dose-escalation study. Cancer Discov.

[71] Hilton J, Gelmon K, Bedard PL (2022). Results of the phase I CCTG IND.231 trial of CX-5461 in patients with advanced solid tumors enriched for DNA-repair deficiencies. Nat Commun.

[72] Moruno-Manchon JF, Koellhoffer EC, Gopakumar J (2017). The G-quadruplex DNA stabilizing drug pyridostatin promotes DNA damage and downregulates transcription of Brca1 in neurons. Aging (Albany NY).

[73] Moruno-Manchon JF, Lejault P, Wang Y (2020). Small-molecule G-quadruplex stabilizers reveal a novel pathway of autophagy regulation in neurons. Elife.

[74] Miglietta G, Russo M, Duardo RC, Capranico G (2021). G-quadruplex binders as cytostatic modulators of innate immune genes in cancer cells. Nucleic Acids Res.

[75] Jiang X-C, Tu F-H, Wei L-Y (2022). Discovery of a novel G-quadruplex and histone deacetylase (HDAC) dual-targeting agent for the treatment of triple-negative breast cancer. J Med Chem.

[76] Krönke J, Hurst SN, Ebert BL (2014). Lenalidomide induces degradation of IKZF1 and IKZF3. OncoImmunology.

[77] Shorstova T, Foulkes WD, Witcher M (2021). Achieving clinical success with BET inhibitors as anti-cancer agents. Br J Cancer.

