# Supplementary material for: Targeting transcription-replication conflicts using G-quadruplexes stabilizers in multiple myeloma
Source: Blood Neoplasia. 2025 Jan 20;2(2):100072. doi: 10.1016/j.bneo.2025.100072 (PMC12067907; doi:10.1016/j.bneo.2025.100072)
Supplement: Supplemental Figures [file BNEO_NEO-2024-000343-mmc2.pptx]

## Slide 1
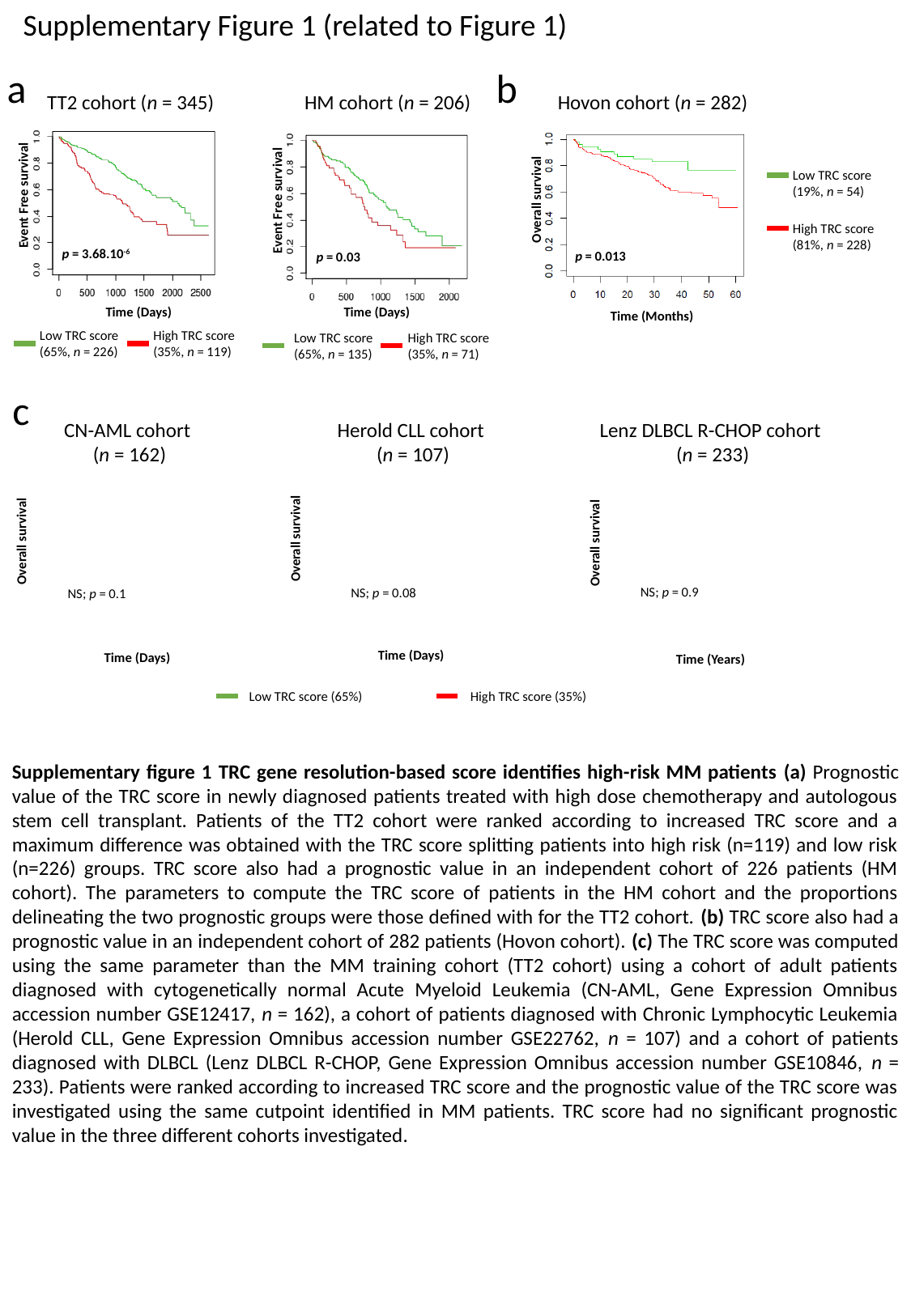

Supplementary Figure 1 (related to Figure 1)
a
b
TT2 cohort (n = 345)
HM cohort (n = 206)
Hovon cohort (n = 282)
Low TRC score
(19%, n = 54)
Event Free survival
Overall survival
Event Free survival
High TRC score
(81%, n = 228)
p = 3.68.10-6
p = 0.013
p = 0.03
Time (Days)
Time (Days)
Time (Months)
Low TRC score (65%, n = 226)
High TRC score (35%, n = 119)
Low TRC score (65%, n = 135)
High TRC score (35%, n = 71)
c
CN-AML cohort
(n = 162)
Herold CLL cohort
(n = 107)
Lenz DLBCL R-CHOP cohort
(n = 233)
Overall survival
Overall survival
Overall survival
NS; p = 0.9
NS; p = 0.08
NS; p = 0.1
Time (Days)
Time (Days)
Time (Years)
Low TRC score (65%)
High TRC score (35%)
Supplementary figure 1 TRC gene resolution-based score identifies high-risk MM patients (a) Prognostic value of the TRC score in newly diagnosed patients treated with high dose chemotherapy and autologous stem cell transplant. Patients of the TT2 cohort were ranked according to increased TRC score and a maximum difference was obtained with the TRC score splitting patients into high risk (n=119) and low risk (n=226) groups. TRC score also had a prognostic value in an independent cohort of 226 patients (HM cohort). The parameters to compute the TRC score of patients in the HM cohort and the proportions delineating the two prognostic groups were those defined with for the TT2 cohort. (b) TRC score also had a prognostic value in an independent cohort of 282 patients (Hovon cohort). (c) The TRC score was computed using the same parameter than the MM training cohort (TT2 cohort) using a cohort of adult patients diagnosed with cytogenetically normal Acute Myeloid Leukemia (CN-AML, Gene Expression Omnibus accession number GSE12417, n = 162), a cohort of patients diagnosed with Chronic Lymphocytic Leukemia (Herold CLL, Gene Expression Omnibus accession number GSE22762, n = 107) and a cohort of patients diagnosed with DLBCL (Lenz DLBCL R-CHOP, Gene Expression Omnibus accession number GSE10846, n = 233). Patients were ranked according to increased TRC score and the prognostic value of the TRC score was investigated using the same cutpoint identified in MM patients. TRC score had no significant prognostic value in the three different cohorts investigated.

## Slide 2
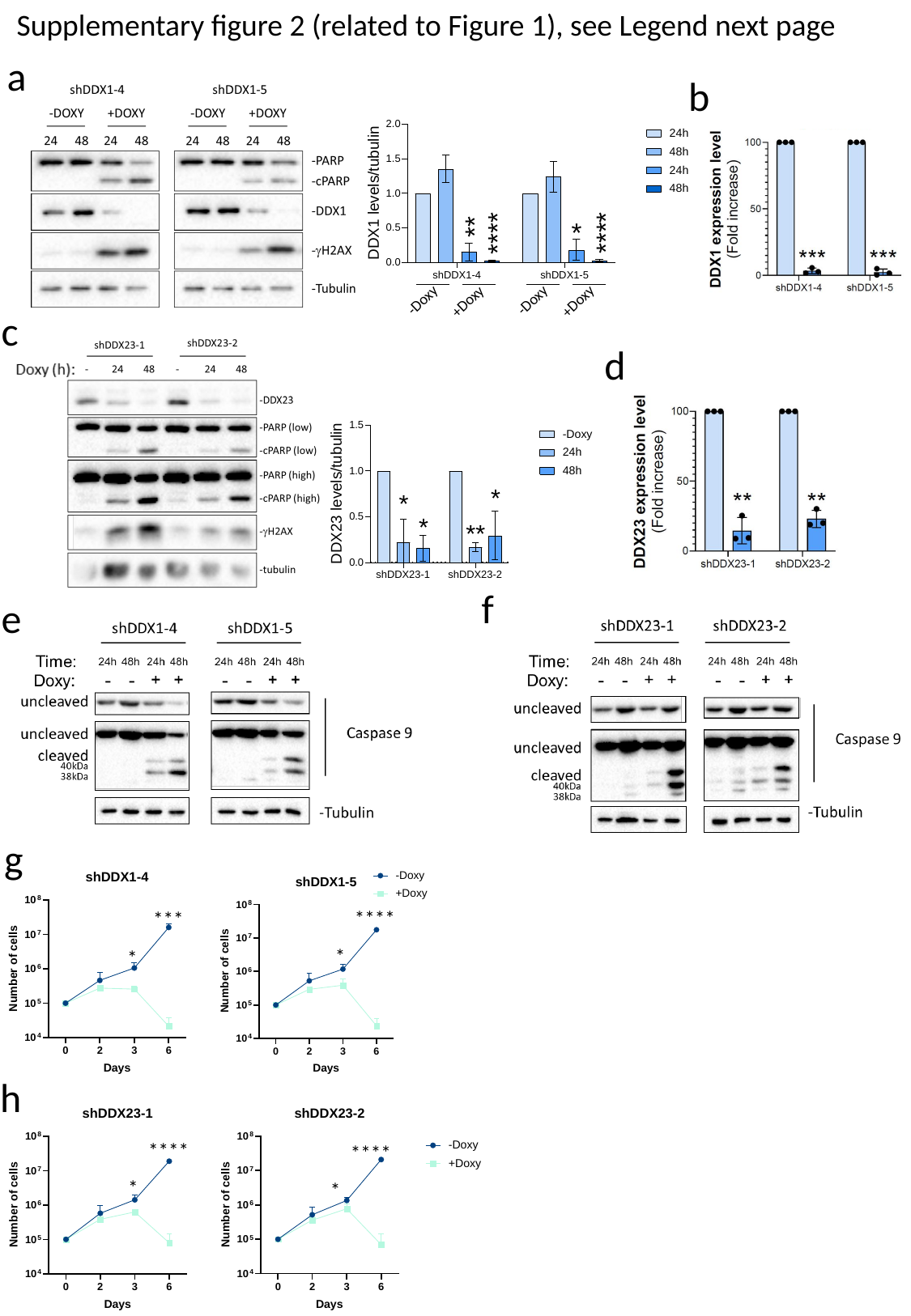

Supplementary figure 2 (related to Figure 1), see Legend next page
c
d
a
b
-Doxy
-Doxy
+Doxy
+Doxy
c
d
f
e
g
****
***
*
*
h
****
****
*
*

## Slide 3
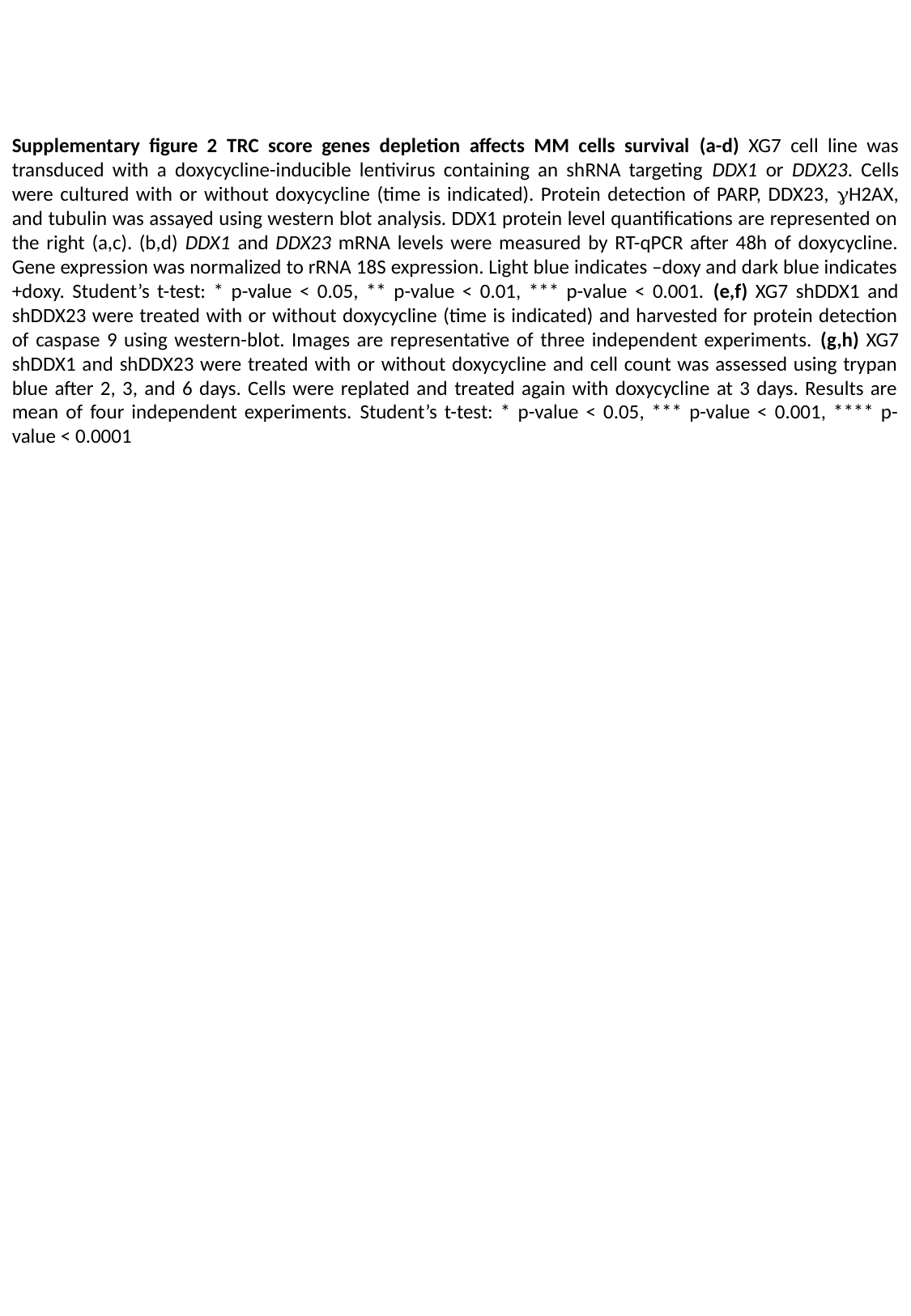

Supplementary figure 2 TRC score genes depletion affects MM cells survival (a-d) XG7 cell line was transduced with a doxycycline-inducible lentivirus containing an shRNA targeting DDX1 or DDX23. Cells were cultured with or without doxycycline (time is indicated). Protein detection of PARP, DDX23, gH2AX, and tubulin was assayed using western blot analysis. DDX1 protein level quantifications are represented on the right (a,c). (b,d) DDX1 and DDX23 mRNA levels were measured by RT-qPCR after 48h of doxycycline. Gene expression was normalized to rRNA 18S expression. Light blue indicates –doxy and dark blue indicates +doxy. Student’s t-test: * p-value < 0.05, ** p-value < 0.01, *** p-value < 0.001. (e,f) XG7 shDDX1 and shDDX23 were treated with or without doxycycline (time is indicated) and harvested for protein detection of caspase 9 using western-blot. Images are representative of three independent experiments. (g,h) XG7 shDDX1 and shDDX23 were treated with or without doxycycline and cell count was assessed using trypan blue after 2, 3, and 6 days. Cells were replated and treated again with doxycycline at 3 days. Results are mean of four independent experiments. Student’s t-test: * p-value < 0.05, *** p-value < 0.001, **** p-value < 0.0001

## Slide 4
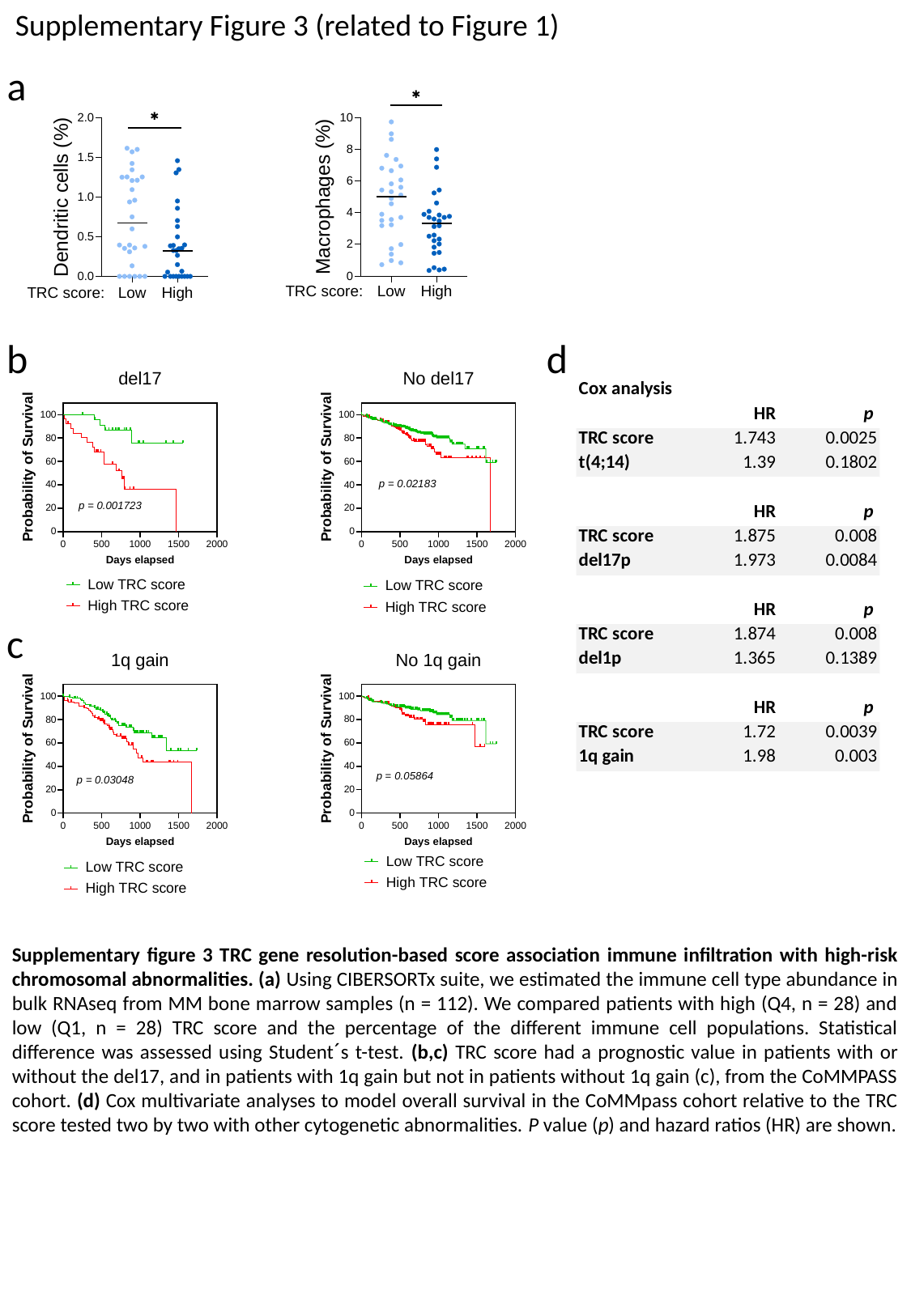

Supplementary Figure 3 (related to Figure 1)
a
b
d
c
Supplementary figure 3 TRC gene resolution-based score association immune infiltration with high-risk chromosomal abnormalities. (a) Using CIBERSORTx suite, we estimated the immune cell type abundance in bulk RNAseq from MM bone marrow samples (n = 112). We compared patients with high (Q4, n = 28) and low (Q1, n = 28) TRC score and the percentage of the different immune cell populations. Statistical difference was assessed using Student ́s t-test. (b,c) TRC score had a prognostic value in patients with or without the del17, and in patients with 1q gain but not in patients without 1q gain (c), from the CoMMPASS cohort. (d) Cox multivariate analyses to model overall survival in the CoMMpass cohort relative to the TRC score tested two by two with other cytogenetic abnormalities. P value (p) and hazard ratios (HR) are shown.

## Slide 5
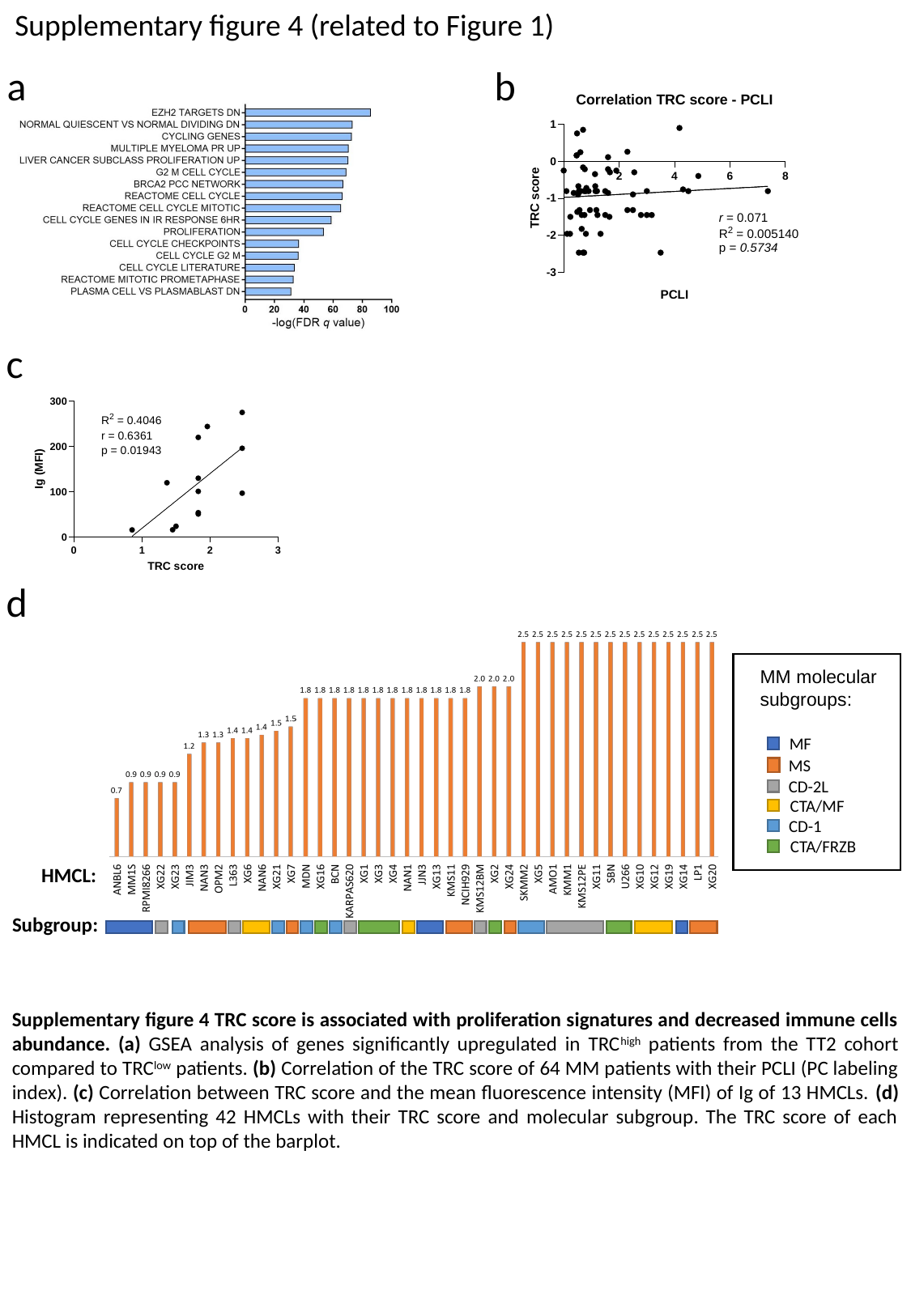

Supplementary figure 4 (related to Figure 1)
a
b
c
d
MM molecular subgroups:
MF
MS
CD-2L
CTA/MF
CD-1
CTA/FRZB
HMCL:
Subgroup:
Supplementary figure 4 TRC score is associated with proliferation signatures and decreased immune cells abundance. (a) GSEA analysis of genes significantly upregulated in TRChigh patients from the TT2 cohort compared to TRClow patients. (b) Correlation of the TRC score of 64 MM patients with their PCLI (PC labeling index). (c) Correlation between TRC score and the mean fluorescence intensity (MFI) of Ig of 13 HMCLs. (d) Histogram representing 42 HMCLs with their TRC score and molecular subgroup. The TRC score of each HMCL is indicated on top of the barplot.

## Slide 6
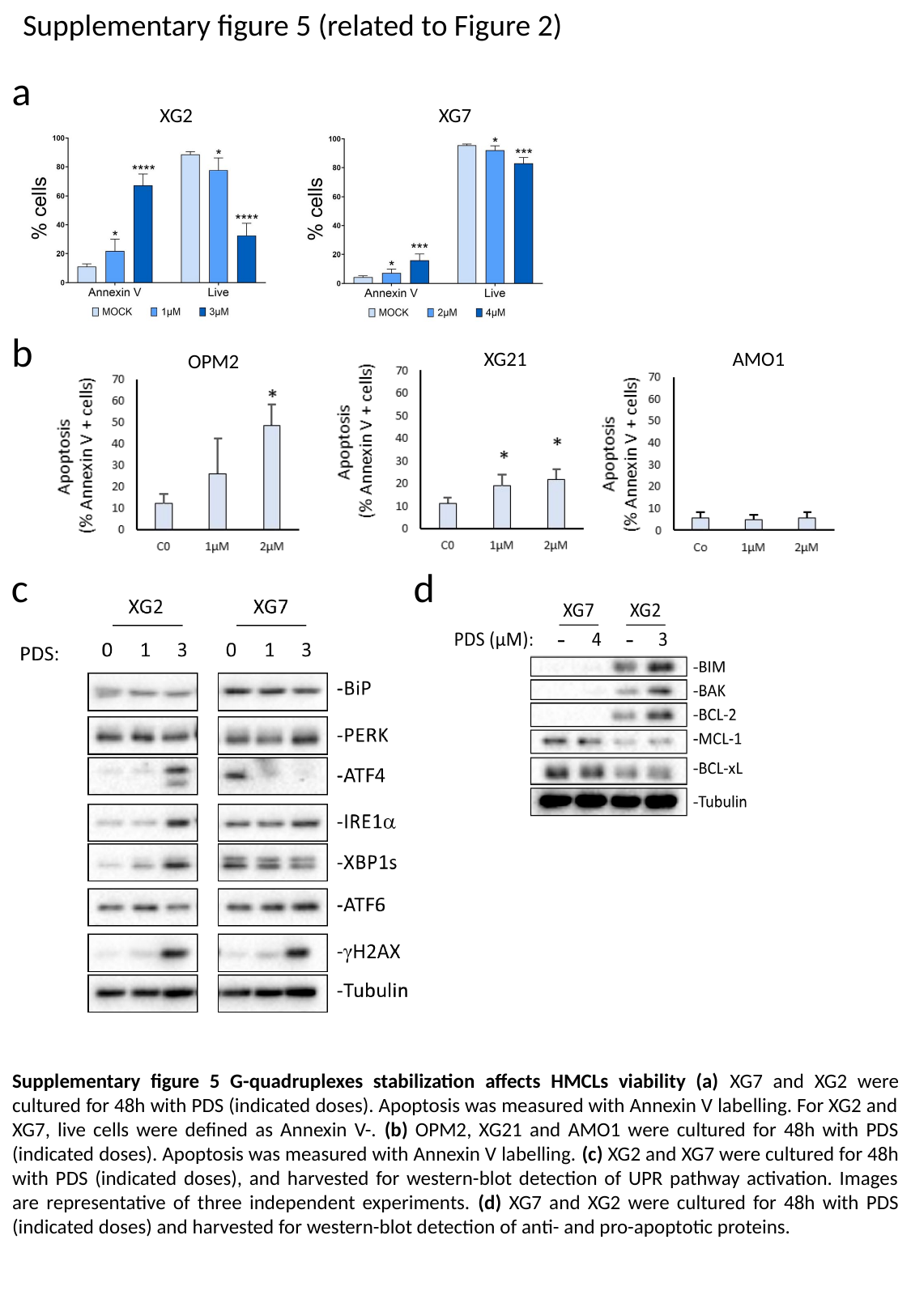

Supplementary figure 5 (related to Figure 2)
a
XG2
XG7
b
XG21
AMO1
OPM2
c
d
Supplementary figure 5 G-quadruplexes stabilization affects HMCLs viability (a) XG7 and XG2 were cultured for 48h with PDS (indicated doses). Apoptosis was measured with Annexin V labelling. For XG2 and XG7, live cells were defined as Annexin V-. (b) OPM2, XG21 and AMO1 were cultured for 48h with PDS (indicated doses). Apoptosis was measured with Annexin V labelling. (c) XG2 and XG7 were cultured for 48h with PDS (indicated doses), and harvested for western-blot detection of UPR pathway activation. Images are representative of three independent experiments. (d) XG7 and XG2 were cultured for 48h with PDS (indicated doses) and harvested for western-blot detection of anti- and pro-apoptotic proteins.

## Slide 7
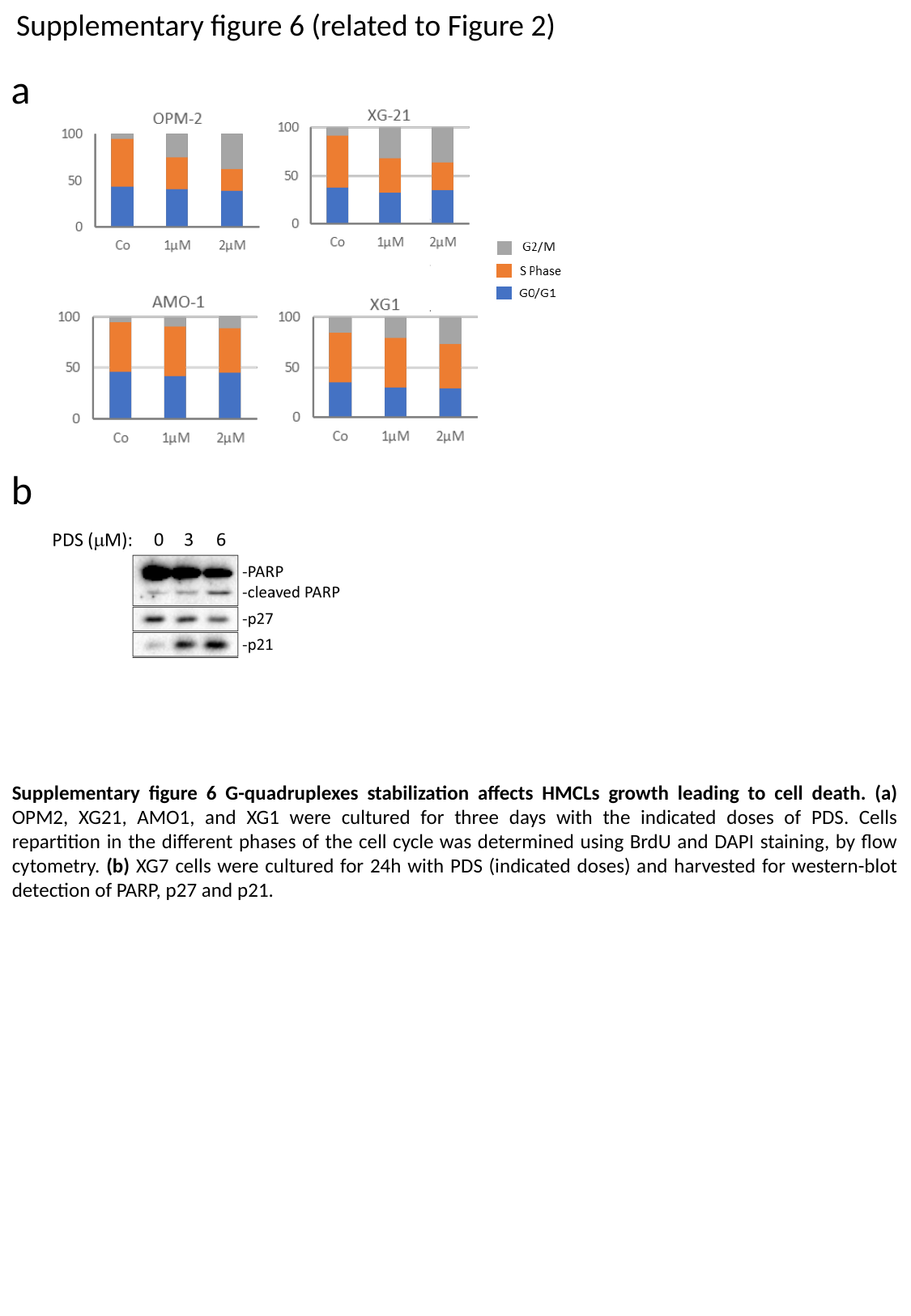

Supplementary figure 6 (related to Figure 2)
a
b
Supplementary figure 6 G-quadruplexes stabilization affects HMCLs growth leading to cell death. (a) OPM2, XG21, AMO1, and XG1 were cultured for three days with the indicated doses of PDS. Cells repartition in the different phases of the cell cycle was determined using BrdU and DAPI staining, by flow cytometry. (b) XG7 cells were cultured for 24h with PDS (indicated doses) and harvested for western-blot detection of PARP, p27 and p21.

## Slide 8
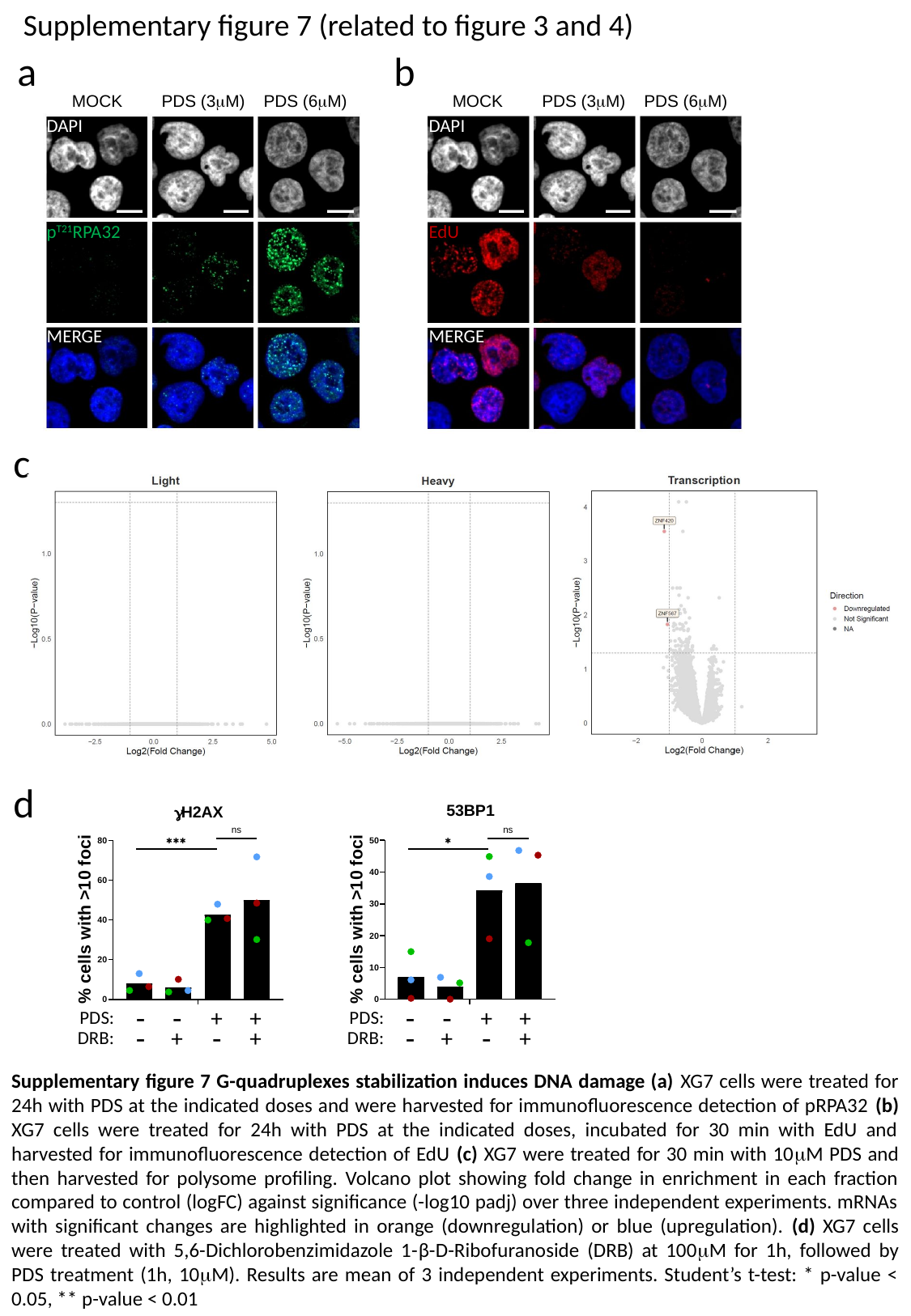

Supplementary figure 7 (related to figure 3 and 4)
a
b
MOCK
PDS (3mM)
PDS (6mM)
MOCK
PDS (3mM)
PDS (6mM)
DAPI
DAPI
pT21RPA32
EdU
MERGE
MERGE
c
d
-
-
+
+
-
-
+
+
-
-
+
+
-
-
+
+
PDS:
PDS:
DRB:
DRB:
Supplementary figure 7 G-quadruplexes stabilization induces DNA damage (a) XG7 cells were treated for 24h with PDS at the indicated doses and were harvested for immunofluorescence detection of pRPA32 (b) XG7 cells were treated for 24h with PDS at the indicated doses, incubated for 30 min with EdU and harvested for immunofluorescence detection of EdU (c) XG7 were treated for 30 min with 10mM PDS and then harvested for polysome profiling. Volcano plot showing fold change in enrichment in each fraction compared to control (logFC) against significance (-log10 padj) over three independent experiments. mRNAs with significant changes are highlighted in orange (downregulation) or blue (upregulation). (d) XG7 cells were treated with 5,6-Dichlorobenzimidazole 1-β-D-Ribofuranoside (DRB) at 100mM for 1h, followed by PDS treatment (1h, 10mM). Results are mean of 3 independent experiments. Student’s t-test: * p-value < 0.05, ** p-value < 0.01

## Slide 9
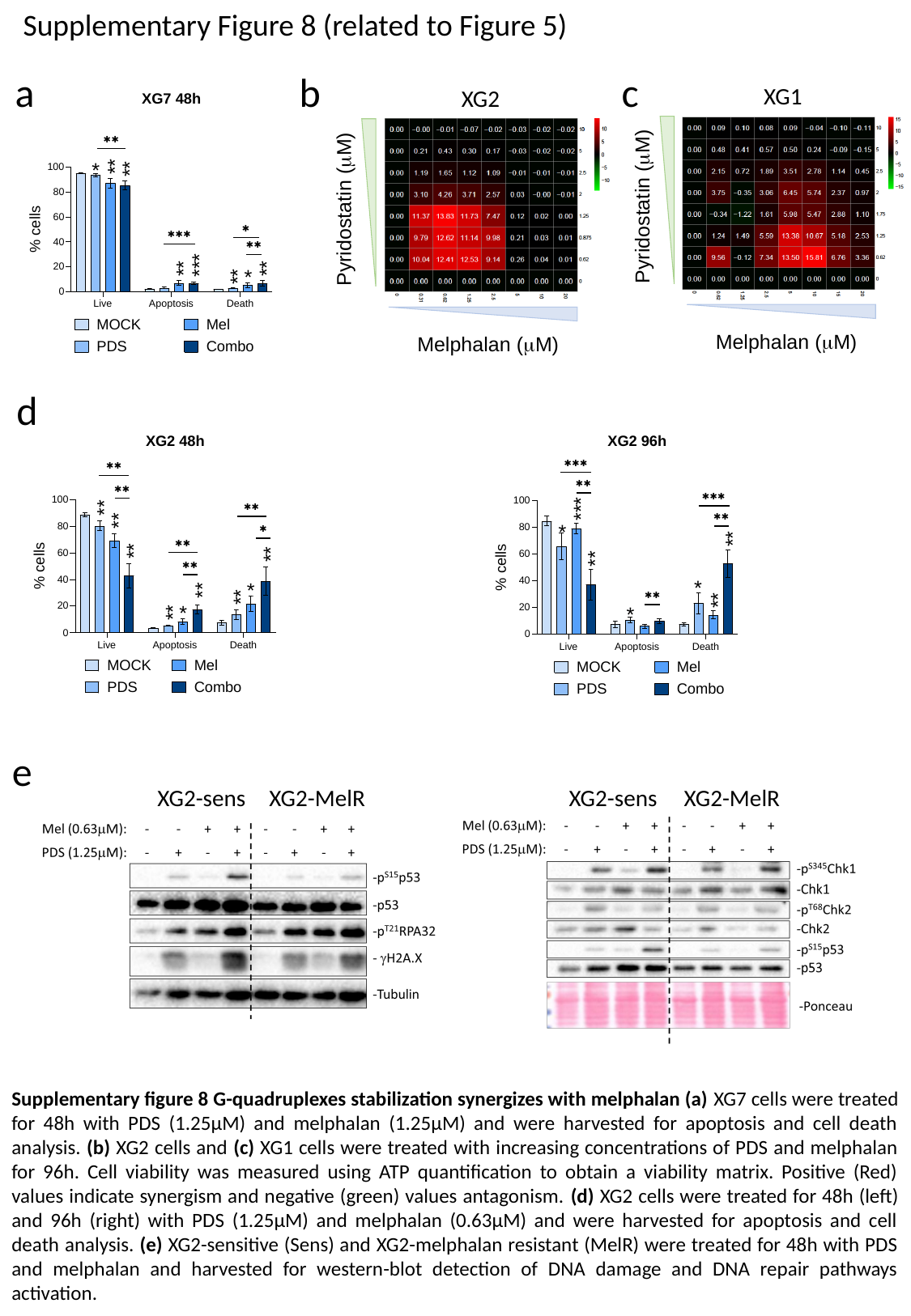

Supplementary Figure 8 (related to Figure 5)
a
b
c
XG1
XG2
Pyridostatin (mM)
Pyridostatin (mM)
 Melphalan (mM)
 Melphalan (mM)
d
e
XG2-sens
XG2-MelR
XG2-sens
XG2-MelR
Supplementary figure 8 G-quadruplexes stabilization synergizes with melphalan (a) XG7 cells were treated for 48h with PDS (1.25µM) and melphalan (1.25µM) and were harvested for apoptosis and cell death analysis. (b) XG2 cells and (c) XG1 cells were treated with increasing concentrations of PDS and melphalan for 96h. Cell viability was measured using ATP quantification to obtain a viability matrix. Positive (Red) values indicate synergism and negative (green) values antagonism. (d) XG2 cells were treated for 48h (left) and 96h (right) with PDS (1.25µM) and melphalan (0.63µM) and were harvested for apoptosis and cell death analysis. (e) XG2-sensitive (Sens) and XG2-melphalan resistant (MelR) were treated for 48h with PDS and melphalan and harvested for western-blot detection of DNA damage and DNA repair pathways activation.

## Slide 10
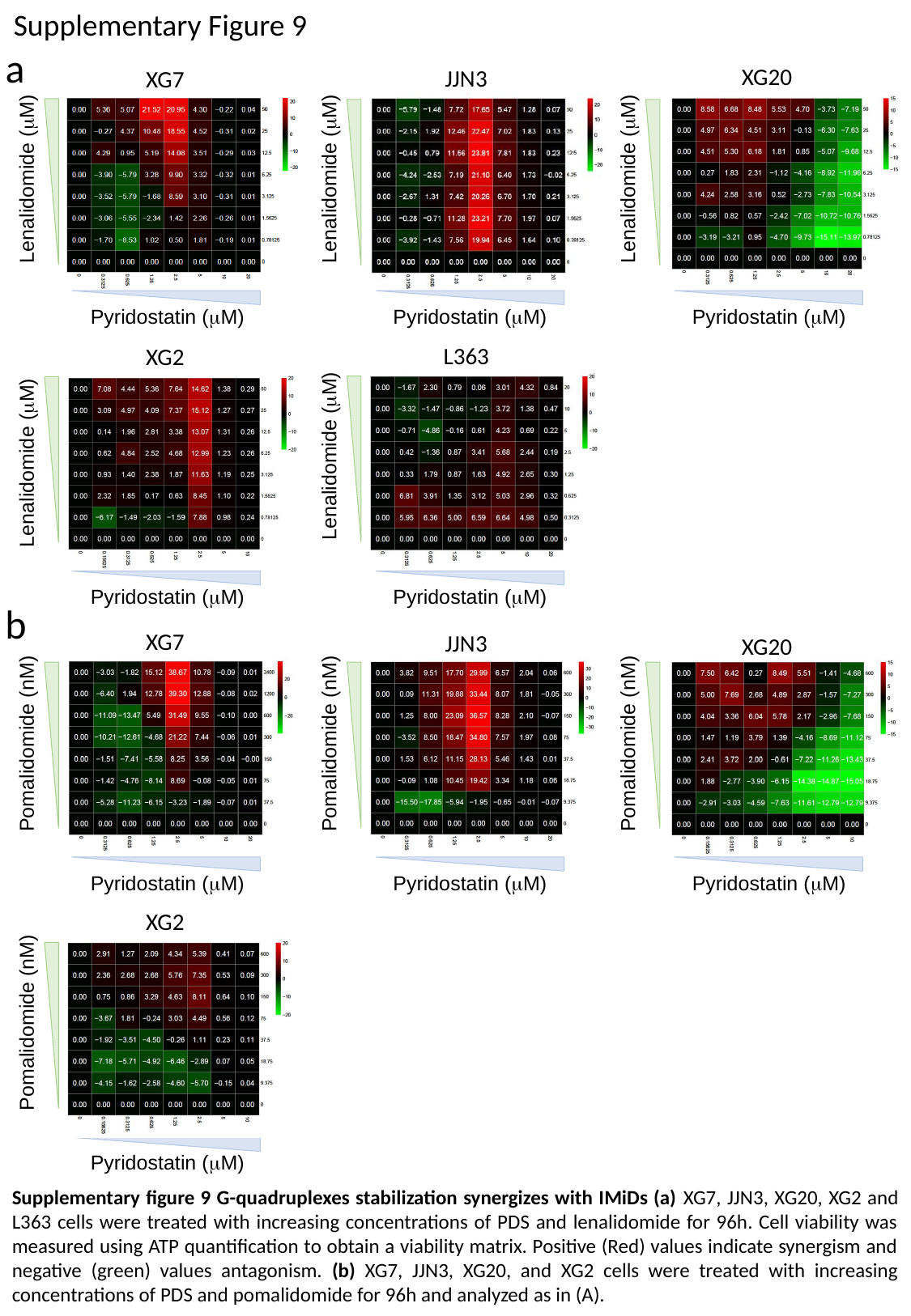

Supplementary Figure 9
a
XG20
JJN3
XG7
 Lenalidomide (mM)
 Lenalidomide (mM)
 Lenalidomide (mM)
Pyridostatin (mM)
Pyridostatin (mM)
Pyridostatin (mM)
L363
XG2
 Lenalidomide (mM)
 Lenalidomide (mM)
Pyridostatin (mM)
Pyridostatin (mM)
b
XG7
JJN3
XG20
 Pomalidomide (nM)
 Pomalidomide (nM)
 Pomalidomide (nM)
Pyridostatin (mM)
Pyridostatin (mM)
Pyridostatin (mM)
XG2
 Pomalidomide (nM)
Pyridostatin (mM)
Supplementary figure 9 G-quadruplexes stabilization synergizes with IMiDs (a) XG7, JJN3, XG20, XG2 and L363 cells were treated with increasing concentrations of PDS and lenalidomide for 96h. Cell viability was measured using ATP quantification to obtain a viability matrix. Positive (Red) values indicate synergism and negative (green) values antagonism. (b) XG7, JJN3, XG20, and XG2 cells were treated with increasing concentrations of PDS and pomalidomide for 96h and analyzed as in (A).

## Slide 11
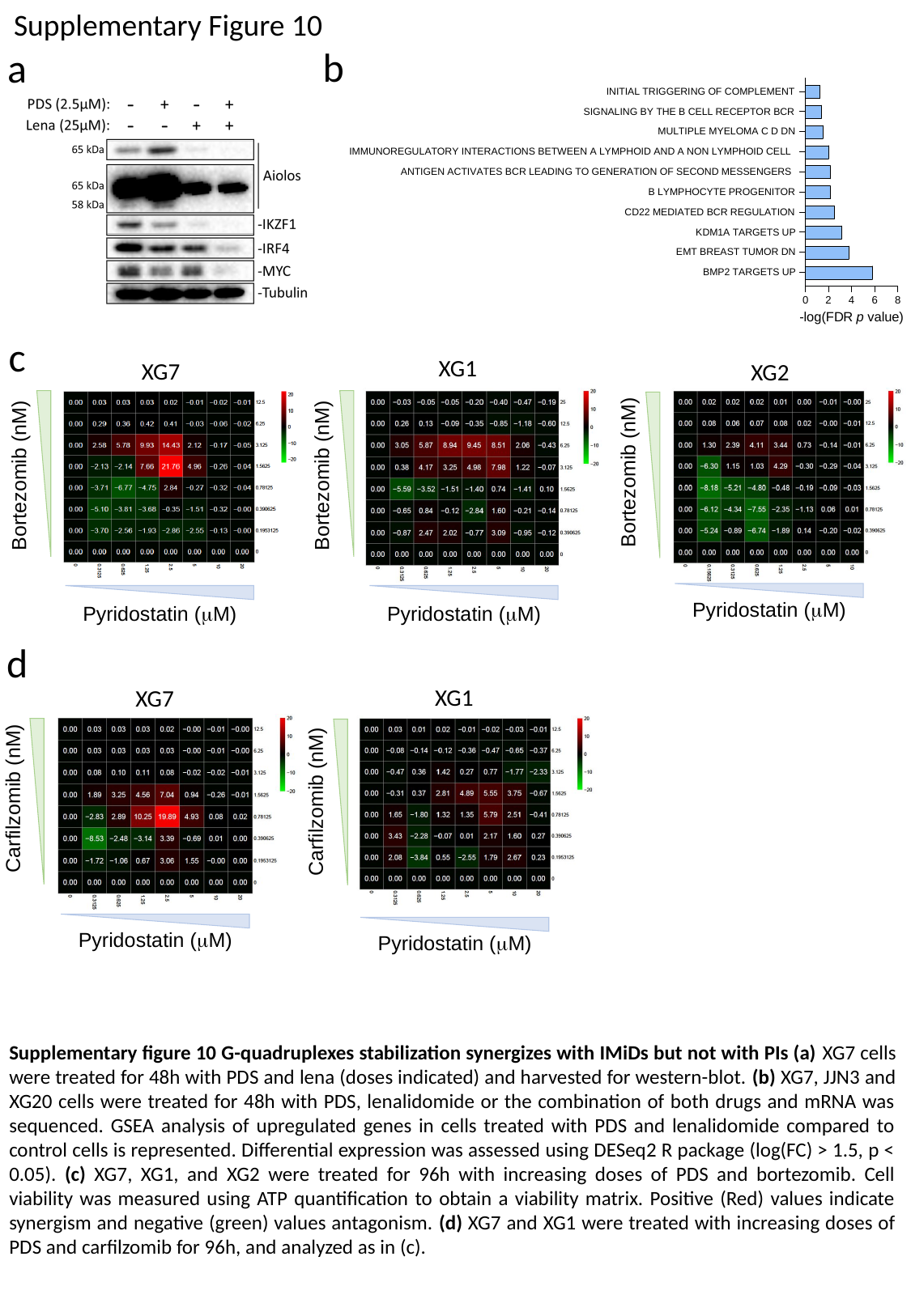

Supplementary Figure 10
b
a
c
XG1
XG7
XG2
 Bortezomib (nM)
 Bortezomib (nM)
 Bortezomib (nM)
Pyridostatin (mM)
Pyridostatin (mM)
Pyridostatin (mM)
d
XG1
XG7
 Carfilzomib (nM)
 Carfilzomib (nM)
Pyridostatin (mM)
Pyridostatin (mM)
Supplementary figure 10 G-quadruplexes stabilization synergizes with IMiDs but not with PIs (a) XG7 cells were treated for 48h with PDS and lena (doses indicated) and harvested for western-blot. (b) XG7, JJN3 and XG20 cells were treated for 48h with PDS, lenalidomide or the combination of both drugs and mRNA was sequenced. GSEA analysis of upregulated genes in cells treated with PDS and lenalidomide compared to control cells is represented. Differential expression was assessed using DESeq2 R package (log(FC) > 1.5, p < 0.05). (c) XG7, XG1, and XG2 were treated for 96h with increasing doses of PDS and bortezomib. Cell viability was measured using ATP quantification to obtain a viability matrix. Positive (Red) values indicate synergism and negative (green) values antagonism. (d) XG7 and XG1 were treated with increasing doses of PDS and carfilzomib for 96h, and analyzed as in (c).

## Slide 12
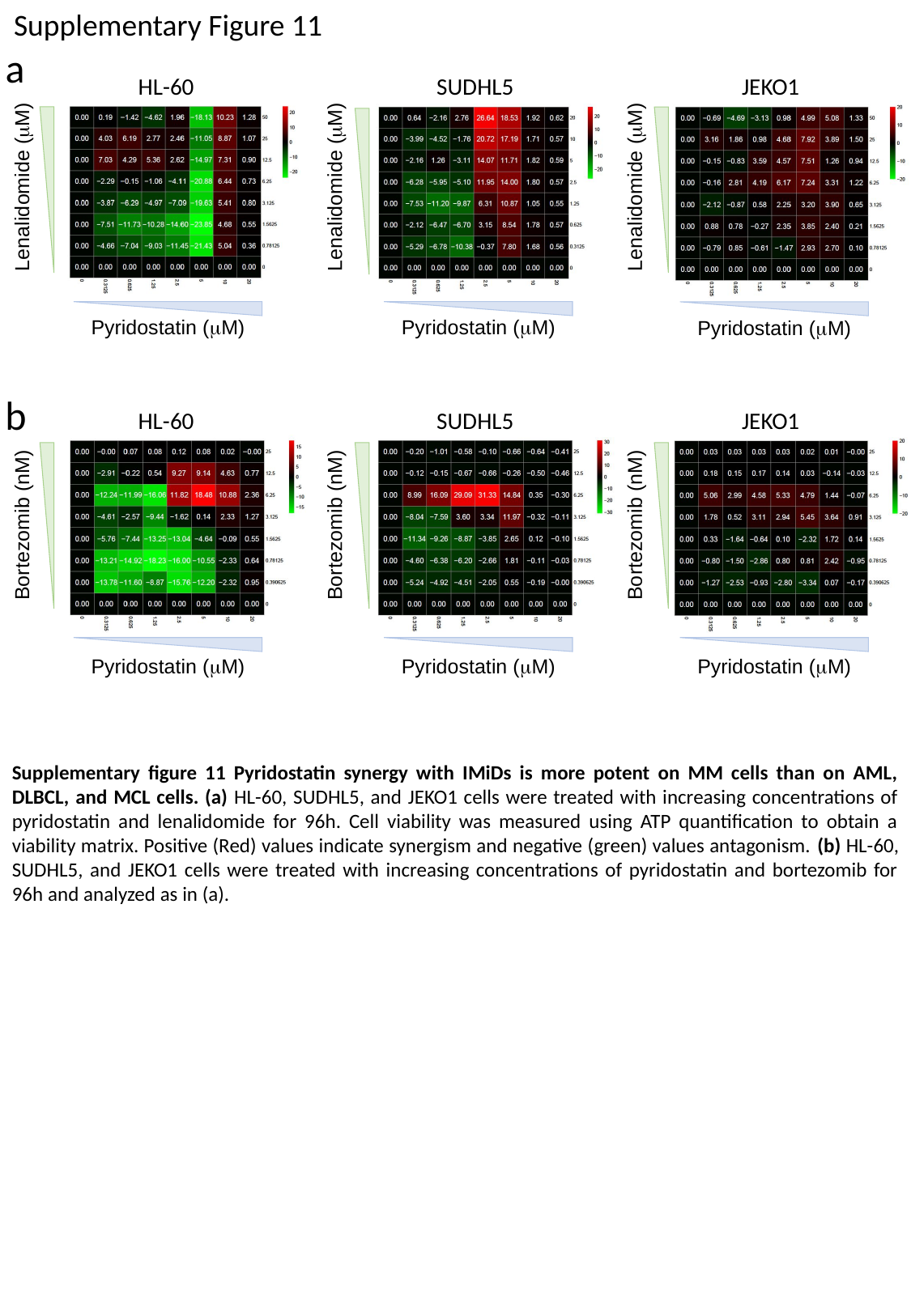

Supplementary Figure 11
a
HL-60
SUDHL5
JEKO1
 Lenalidomide (mM)
 Lenalidomide (mM)
 Lenalidomide (mM)
Pyridostatin (mM)
Pyridostatin (mM)
Pyridostatin (mM)
b
HL-60
SUDHL5
JEKO1
 Bortezomib (nM)
 Bortezomib (nM)
 Bortezomib (nM)
Pyridostatin (mM)
Pyridostatin (mM)
Pyridostatin (mM)
Supplementary figure 11 Pyridostatin synergy with IMiDs is more potent on MM cells than on AML, DLBCL, and MCL cells. (a) HL-60, SUDHL5, and JEKO1 cells were treated with increasing concentrations of pyridostatin and lenalidomide for 96h. Cell viability was measured using ATP quantification to obtain a viability matrix. Positive (Red) values indicate synergism and negative (green) values antagonism. (b) HL-60, SUDHL5, and JEKO1 cells were treated with increasing concentrations of pyridostatin and bortezomib for 96h and analyzed as in (a).

## Slide 13
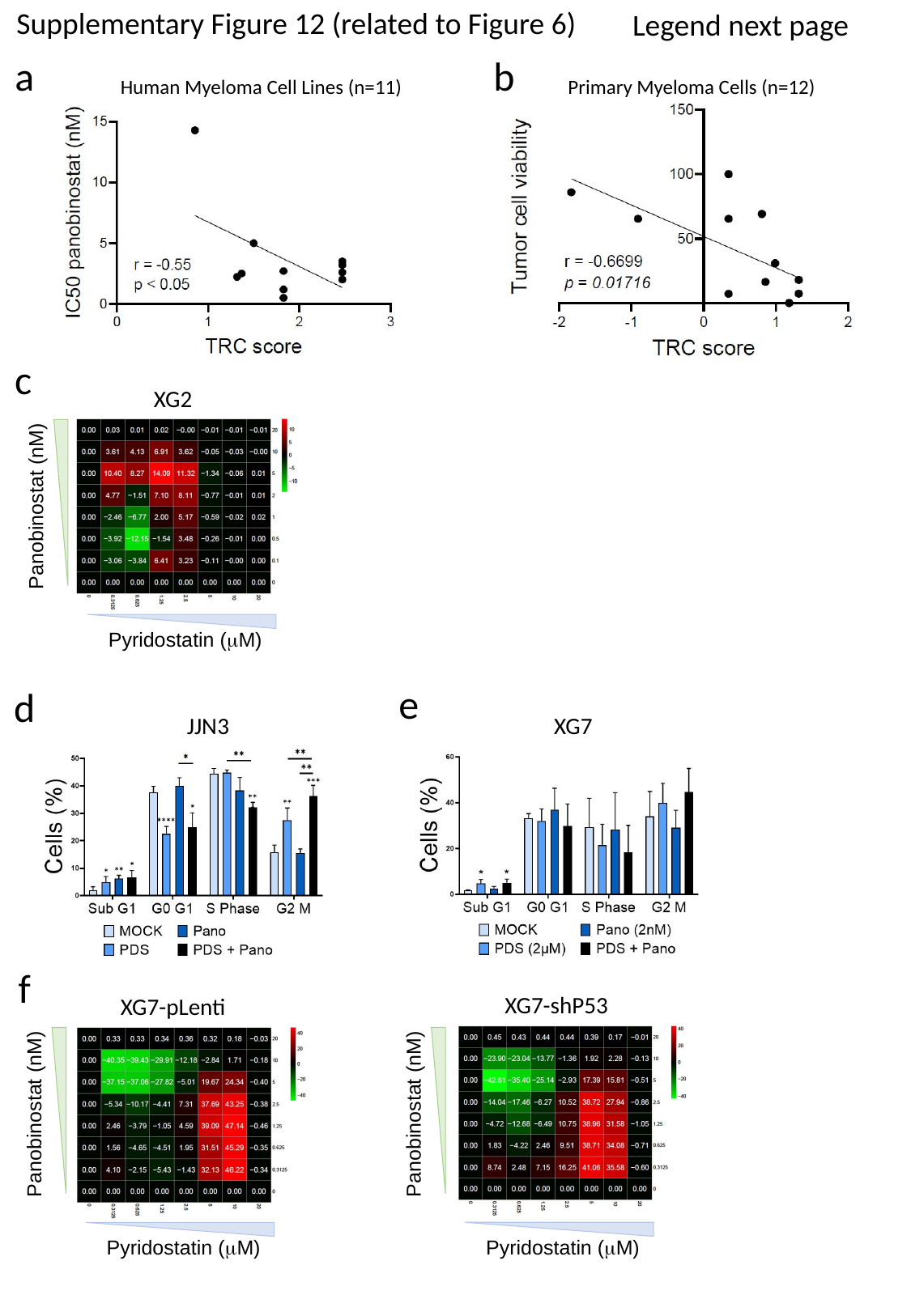

Supplementary Figure 12 (related to Figure 6)
Legend next page
a
b
Human Myeloma Cell Lines (n=11)
Primary Myeloma Cells (n=12)
c
XG2
Panobinostat (nM)
 Pyridostatin (mM)
e
d
JJN3
XG7
f
XG7-shP53
XG7-pLenti
Panobinostat (nM)
Panobinostat (nM)
 Pyridostatin (mM)
 Pyridostatin (mM)

## Slide 14
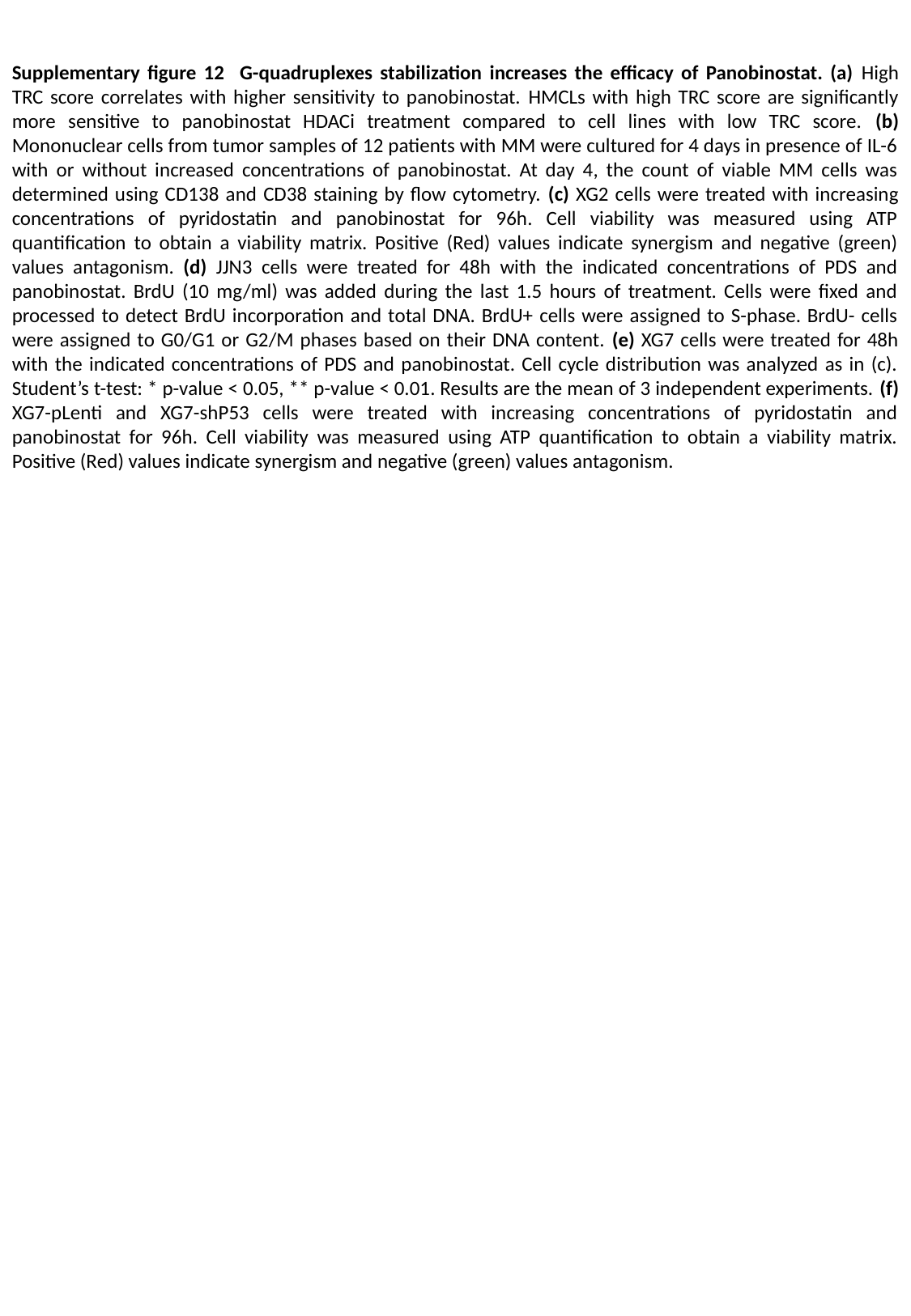

Supplementary figure 12 G-quadruplexes stabilization increases the efficacy of Panobinostat. (a) High TRC score correlates with higher sensitivity to panobinostat. HMCLs with high TRC score are significantly more sensitive to panobinostat HDACi treatment compared to cell lines with low TRC score. (b) Mononuclear cells from tumor samples of 12 patients with MM were cultured for 4 days in presence of IL-6 with or without increased concentrations of panobinostat. At day 4, the count of viable MM cells was determined using CD138 and CD38 staining by flow cytometry. (c) XG2 cells were treated with increasing concentrations of pyridostatin and panobinostat for 96h. Cell viability was measured using ATP quantification to obtain a viability matrix. Positive (Red) values indicate synergism and negative (green) values antagonism. (d) JJN3 cells were treated for 48h with the indicated concentrations of PDS and panobinostat. BrdU (10 mg/ml) was added during the last 1.5 hours of treatment. Cells were fixed and processed to detect BrdU incorporation and total DNA. BrdU+ cells were assigned to S-phase. BrdU- cells were assigned to G0/G1 or G2/M phases based on their DNA content. (e) XG7 cells were treated for 48h with the indicated concentrations of PDS and panobinostat. Cell cycle distribution was analyzed as in (c). Student’s t-test: * p-value < 0.05, ** p-value < 0.01. Results are the mean of 3 independent experiments. (f) XG7-pLenti and XG7-shP53 cells were treated with increasing concentrations of pyridostatin and panobinostat for 96h. Cell viability was measured using ATP quantification to obtain a viability matrix. Positive (Red) values indicate synergism and negative (green) values antagonism.

## Slide 15
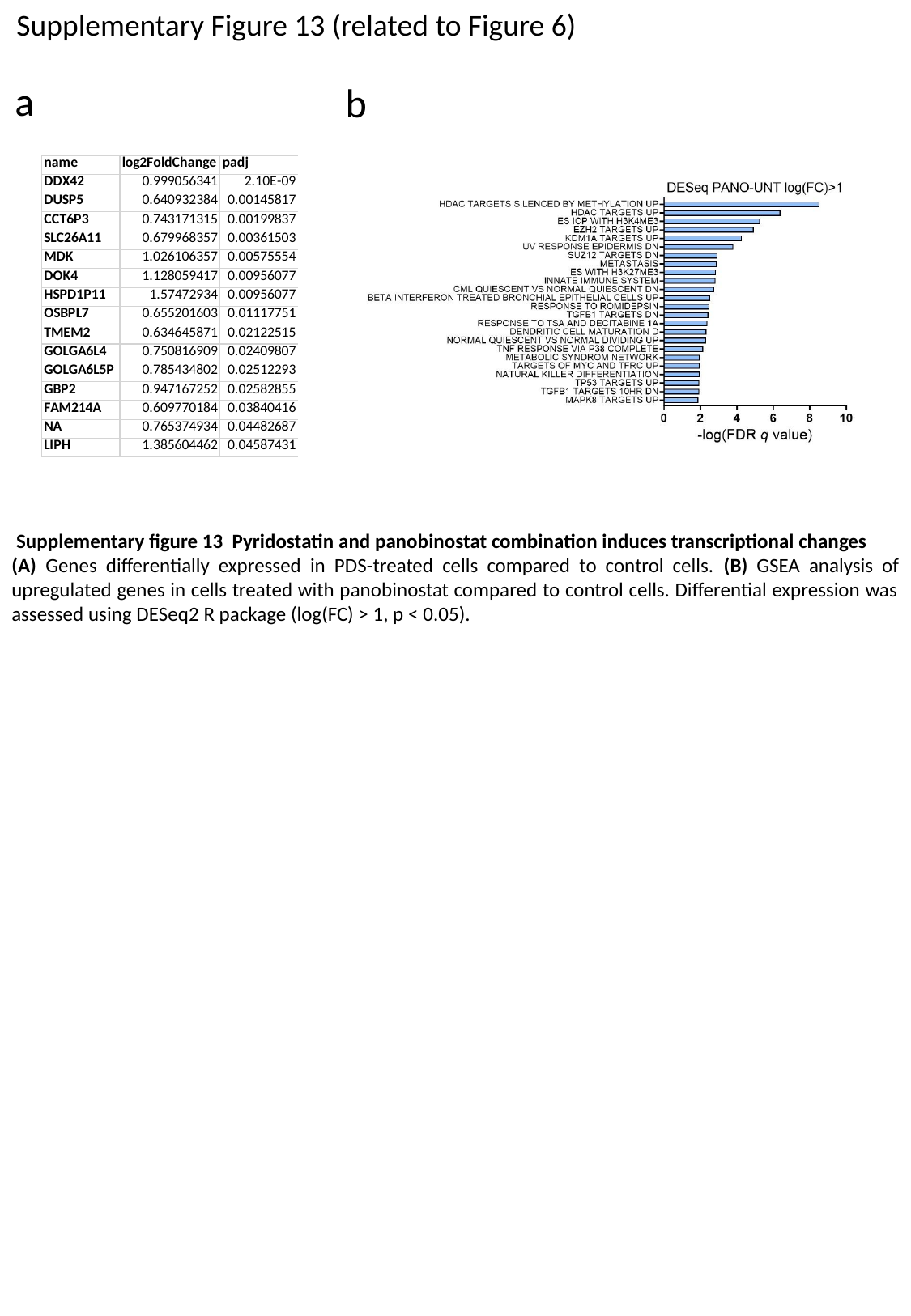

Supplementary Figure 13 (related to Figure 6)
a
b
 Supplementary figure 13 Pyridostatin and panobinostat combination induces transcriptional changes
(A) Genes differentially expressed in PDS-treated cells compared to control cells. (B) GSEA analysis of upregulated genes in cells treated with panobinostat compared to control cells. Differential expression was assessed using DESeq2 R package (log(FC) > 1, p < 0.05).

## Slide 16
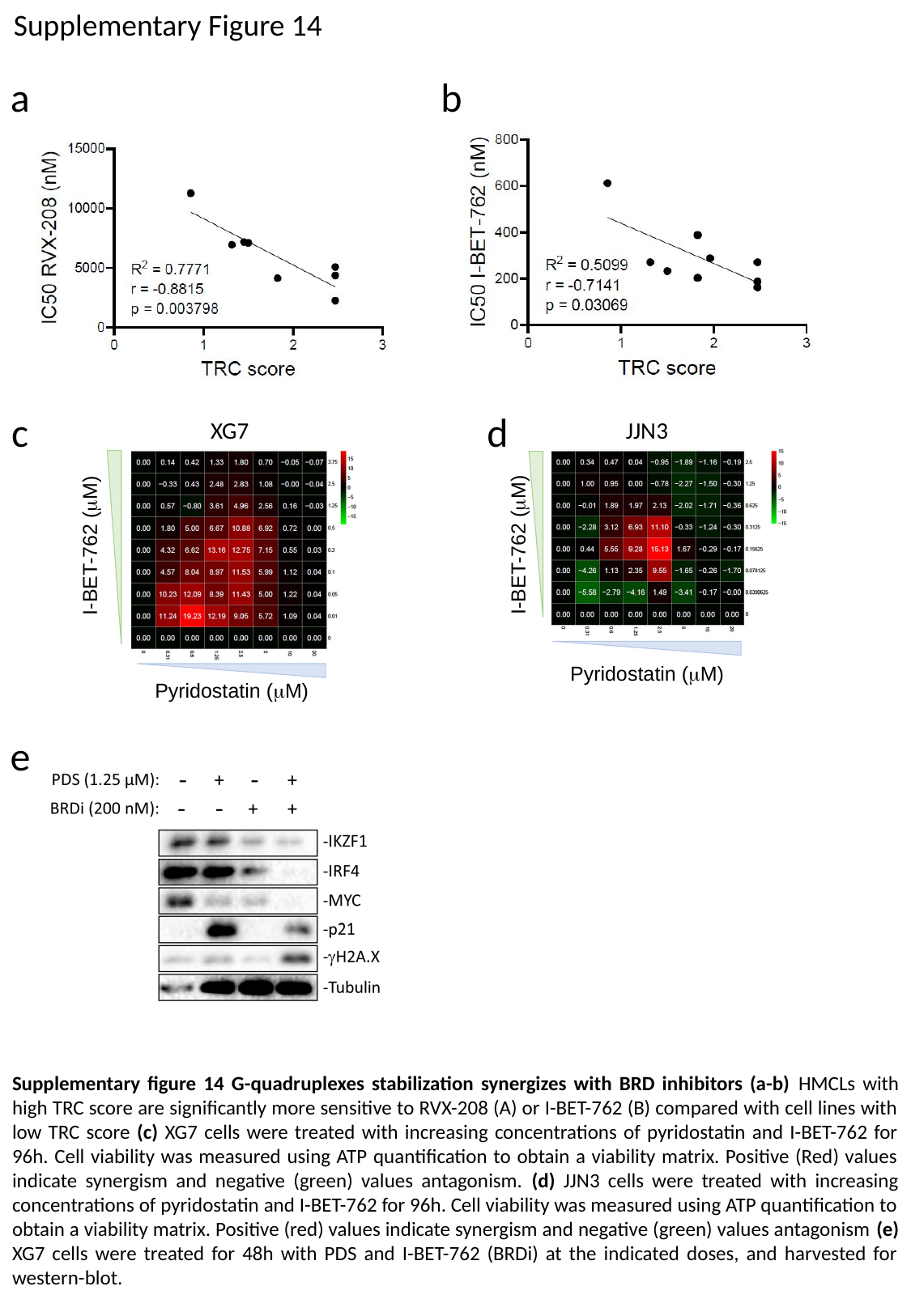

Supplementary Figure 14
a
b
c
d
JJN3
XG7
I-BET-762 (mM)
I-BET-762 (mM)
 Pyridostatin (mM)
 Pyridostatin (mM)
e
Supplementary figure 14 G-quadruplexes stabilization synergizes with BRD inhibitors (a-b) HMCLs with high TRC score are significantly more sensitive to RVX-208 (A) or I-BET-762 (B) compared with cell lines with low TRC score (c) XG7 cells were treated with increasing concentrations of pyridostatin and I-BET-762 for 96h. Cell viability was measured using ATP quantification to obtain a viability matrix. Positive (Red) values indicate synergism and negative (green) values antagonism. (d) JJN3 cells were treated with increasing concentrations of pyridostatin and I-BET-762 for 96h. Cell viability was measured using ATP quantification to obtain a viability matrix. Positive (red) values indicate synergism and negative (green) values antagonism (e) XG7 cells were treated for 48h with PDS and I-BET-762 (BRDi) at the indicated doses, and harvested for western-blot.

## Slide 17
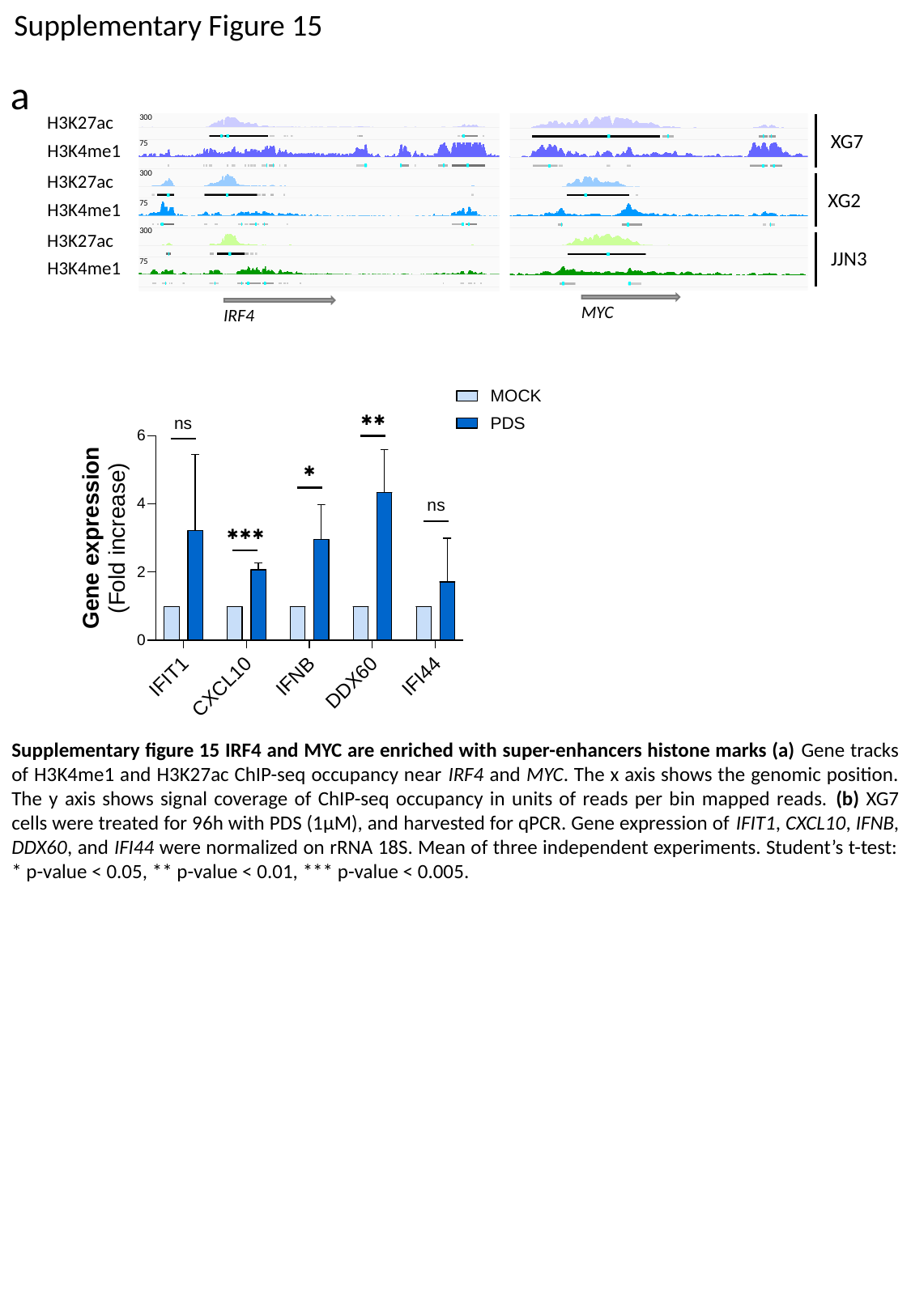

Supplementary Figure 15
a
H3K27ac
300
XG7
75
H3K4me1
300
H3K27ac
XG2
75
H3K4me1
300
H3K27ac
JJN3
75
H3K4me1
MYC
IRF4
Supplementary figure 15 IRF4 and MYC are enriched with super-enhancers histone marks (a) Gene tracks of H3K4me1 and H3K27ac ChIP-seq occupancy near IRF4 and MYC. The x axis shows the genomic position. The y axis shows signal coverage of ChIP-seq occupancy in units of reads per bin mapped reads. (b) XG7 cells were treated for 96h with PDS (1µM), and harvested for qPCR. Gene expression of IFIT1, CXCL10, IFNB, DDX60, and IFI44 were normalized on rRNA 18S. Mean of three independent experiments. Student’s t-test: * p-value < 0.05, ** p-value < 0.01, *** p-value < 0.005.

## Slide 18
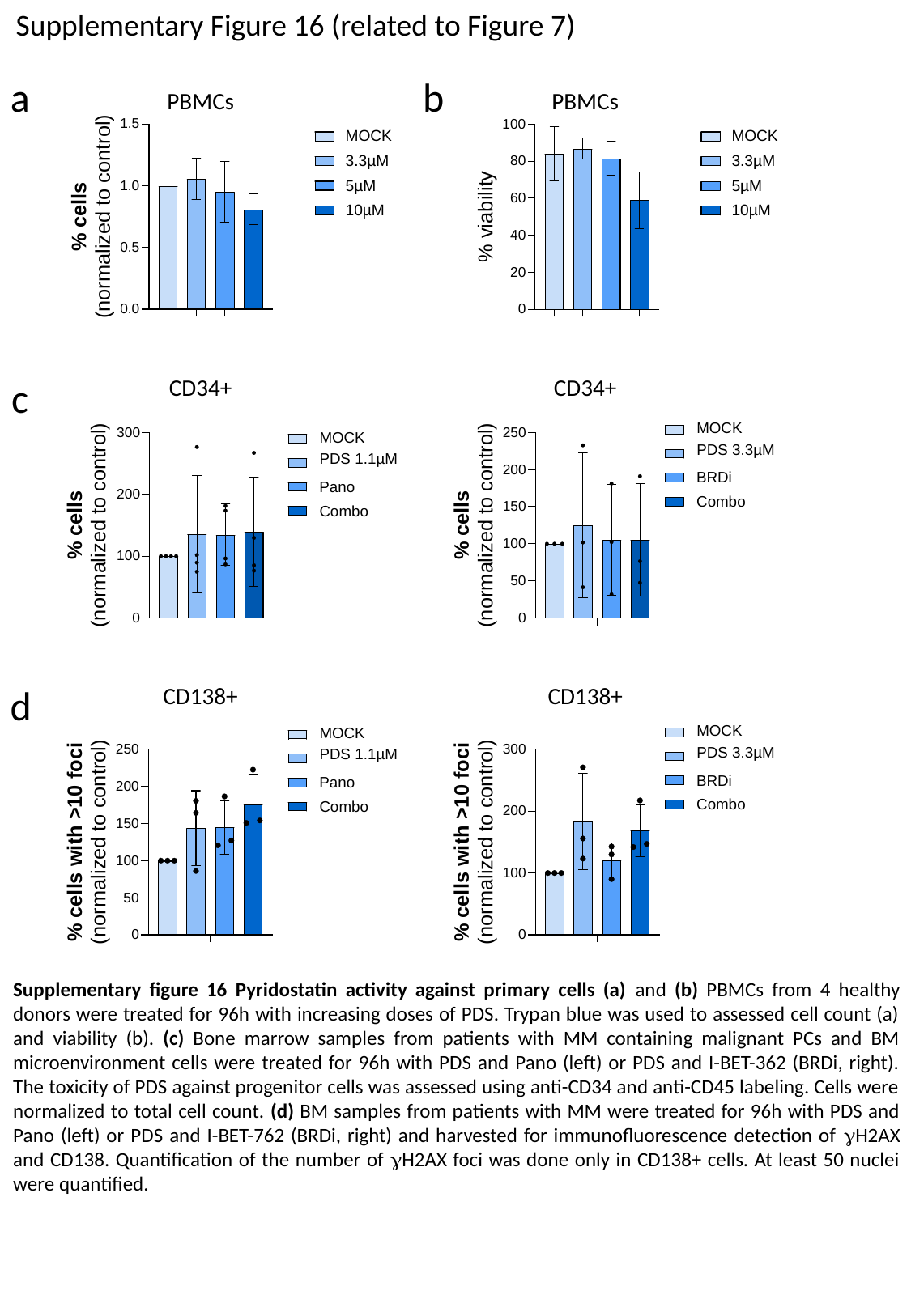

Supplementary Figure 16 (related to Figure 7)
a
b
PBMCs
PBMCs
c
CD34+
CD34+
MOCK
MOCK
PDS 3.3µM
PDS 1.1µM
BRDi
Pano
Combo
Combo
d
CD138+
CD138+
MOCK
MOCK
PDS 3.3µM
PDS 1.1µM
BRDi
Pano
Combo
Combo
Supplementary figure 16 Pyridostatin activity against primary cells (a) and (b) PBMCs from 4 healthy donors were treated for 96h with increasing doses of PDS. Trypan blue was used to assessed cell count (a) and viability (b). (c) Bone marrow samples from patients with MM containing malignant PCs and BM microenvironment cells were treated for 96h with PDS and Pano (left) or PDS and I-BET-362 (BRDi, right). The toxicity of PDS against progenitor cells was assessed using anti-CD34 and anti-CD45 labeling. Cells were normalized to total cell count. (d) BM samples from patients with MM were treated for 96h with PDS and Pano (left) or PDS and I-BET-762 (BRDi, right) and harvested for immunofluorescence detection of gH2AX and CD138. Quantification of the number of gH2AX foci was done only in CD138+ cells. At least 50 nuclei were quantified.
